# Opa1 and Drp1 reciprocally regulate cristae morphology, ETC function, and NAD^+^ regeneration in KRas-mutant lung adenocarcinoma

**DOI:** 10.1016/j.celrep.2022.111818

**Published:** 2022-12-13

**Authors:** Dane T. Sessions, Kee-Beom Kim, Jennifer A. Kashatus, Nikolas Churchill, Kwon-Sik Park, Marty W. Mayo, Hiromi Sesaki, David F. Kashatus

**Affiliations:** 1 Department of Microbiology, Immunology, and Cancer Biology, University of Virginia Health System, Charlottesville, VA 22908, USA; 2 Department of Biochemistry and Molecular Genetics, University of Virginia Health System, Charlottesville, VA 22908, USA; 3 Department of Cell Biology, Johns Hopkins University School of Medicine, Baltimore, MD 21205, USA; 4 Lead contact

## Abstract

Oncogenic KRas activates mitochondrial fission through Erk-mediated phosphorylation of the mitochondrial fission GTPase Drp1. Drp1 deletion inhibits tumorigenesis of KRas-driven pancreatic cancer, but the role of mitochondrial dynamics in other Ras-driven malignancies is poorly defined. Here we show that *in vitro* and *in vivo* growth of KRas-driven lung adenocarcinoma is unaffected by deletion of Drp1 but is inhibited by deletion of Opa1, the GTPase that regulates inner membrane fusion and proper cristae morphology. Mechanistically, Opa1 knockout disrupts cristae morphology and inhibits electron transport chain (ETC) assembly and activity, which inhibits tumor cell proliferation through loss of NAD^+^ regeneration. Simultaneous inactivation of Drp1 and Opa1 restores cristae morphology, ETC activity, and cell proliferation indicating that mitochondrial fission activity drives ETC dysfunction induced by Opa1 knockout. Our results support a model in which mitochondrial fission events disrupt cristae structure, and tumor cells with hyperactive fission activity require Opa1 activity to maintain ETC function.

## In brief

Sessions et al. find that KRas-mutant lung adenocarcinoma requires the mitochondrial fusion GTPase Opa1 to maintain cristae structure and ETC function. Opa1 deletion leads to mitochondrial fission-mediated ETC disassembly and inhibition of NAD^+^ regeneration required for oxidative metabolism. Drp1 deletion does not inhibit LUAD development, but it rescues effects of Opa1 deletion.

## Graphical Abstract

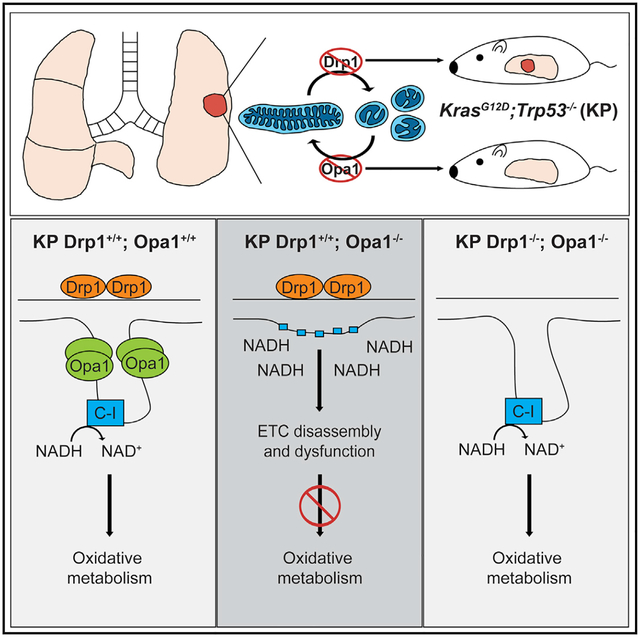

## INTRODUCTION

Approximately one-third of all human tumors harbor mutations in *RAS* GTPases. Activating *RAS* mutations rewires cellular metabolism^1–3^ and promotes cell survival and proliferation. Mitochondria regulate many processes disrupted in cancer, including ATP synthesis, redox homeostasis, and apoptosis. Mitochondria undergo cycles of fusion and fission regulated by four large dynamin-related GTPases. Drp1 (*DNM1L*) executes mitochondrial fission, whereas Mfn1/2 and Opa1 execute fusion of mitochondrial outer and inner membranes, respectively. The fission and fusion GTPases also remodel the mitochondrial outer and inner membranes. For example, Drp1 remodels cristae during apoptosis,^4^ and Opa1 is critical to maintain cristae fidelity^5^ to support oxidative phosphorylation (OXPHOS)^6–8^ and resistance to apoptosis^9,10^ through sequestration of cytochrome C.^11^

Oncogenic signaling impacts mitochondrial shape by regulating mitochondrial dynamics machinery. Oncogenic KRas activates mitochondrial fission through Erk2-mediated phosphorylation of Drp1,^12,13^ and inhibition of mitochondrial fission inhibits KRas-driven glycolytic flux, cellular transformation, and pancreatic tumor (PDAC) growth.^14^ To determine if mitochondrial fission contributes to tumor growth in other Ras-driven malignancies, we explored the effects of mitochondrial dynamics disruption in KRas-driven lung adenocarcinoma (LUAD). LUAD has the highest mortality of human cancers, and 30% of LUAD tumors harbor oncogenic *KRAS* mutations; however, the role of mitochondrial dynamics in this malignancy is poorly defined compared with other KRas-driven tumors. As observed in PDAC and melanoma, Drp1 is phosphorylated downstream of Ras signaling in LUAD and regulates LUAD metabolism.^15,16^ However, while some studies find elevated Drp1 expression in tumors and a pro-tumorigenic role,^15–17^ others demonstrate decreased tumor Drp1 expression and anti-tumor roles.^18^

Tissue of origin influences metabolism of tumors with identical driver mutations.^19,20^ Since PDAC and LUAD exhibit distinct metabolic phenotypes,^21–26^ we explored whether LUAD demonstrates unique sensitivities to dynamics disruption. Surprisingly, deletion of Opa1, but not Drp1, inhibits *in vitro* colony formation and electron transport chain (ETC) function of KRas-driven LUAD cells and blocks lung tumor development in a *Kras*^*LSL-G12D/+*^*; Trp53*^*FL/FL*^ (KP) genetically engineered mouse model (GEMM). Further, Drp1 deletion rescues the effects of Opa1 deletion on *in vitro* colony formation and ETC function but not *in vivo* tumor growth. Mechanistically, Opa1 deletion impacts colony formation and tumor growth through disruption of cristae morphology and inhibition of ETC assembly and function. Consistent with recent work,^27–29^ the critical ETC function disrupted by Opa1 deletion is not mitochondrial ATP synthesis, but rather complex I-mediated NAD^+^ regeneration. Collectively, these data suggest that Opa1-dependent cristae remodeling is critical to complex I-mediated NAD^+^ regeneration in proliferative cells with substantial mitochondrial fission activity.

## RESULTS

### Opa1 inhibition prevents KRas-mutant LUAD colony formation in a Drp1-dependent manner

**Figure 1.**
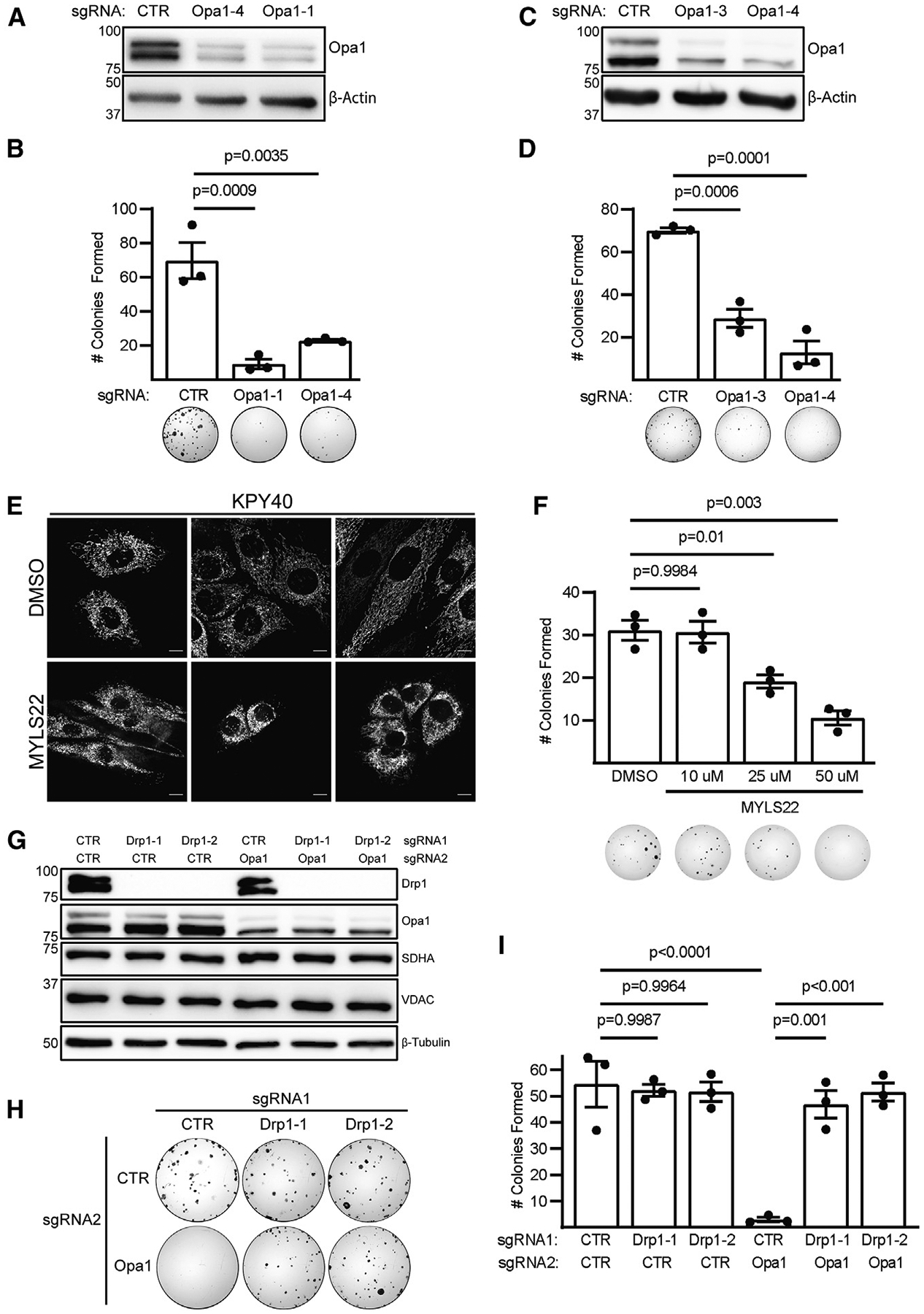
Opa1 inhibition prevents KRas-mutant LUAD colony formation in a Drp1-dependent manner (A) Immunoblot of Opa1 in KPY40 CRISPR cells. (B) Colony formation in KPY40 CRISPR cells with quantification. n = 3 independent experiments. Mean ± SD. One-way ANOVA + Dunnett’s multiple comparisons test. (C) Immunoblot of Opa1 in A549 CRISPR cells. (D) Colony formation in A549 CRISPR cells with quantification. n = 3 independent experiments. Mean ± SD. One-way ANOVA + Dunnett’s multiple comparisons test. (E) Mitotracker green-stained KPY40 tumor cells treated with DMSO or 50 μM MYLS22 for 72 h. Scale represents 10 μm. (F) Representative colony formation in MYLS22-treated KPY40 cells with quantification. n = 3 independent experiments. Mean ± SD. One-way ANOVA + Sidak’s multiple comparisons test. (G) Immunoblot of SDHA, VDAC, Drp1, and Opa1 in KPY40 CRISPR cells. (H) Colony formation in KPY40 double-CRISPR cells. (I) Quantification of KPY40 double-CRISPR colony formation. n = 3 independent experiments. Mean ± SD. One-way ANOVA + Sidak’s multiple comparisons test.

Mitochondrial shape is maintained through a balance of fission and fusion. Persistent Ras-MAPK signaling hyperactivates mitochondrial fission through Erk2-mediated phosphorylation of Drp1 to promote tumorigenesis.^12–14^ We hypothesized that KRas-mutant LUAD may be sensitive to unopposed MAPK-stimulated fission following inhibition of mitochondrial fusion. We used CRISPR-mediated Opa1 deletion to inactivate mitochondrial fusion in KPY40 lung tumor cells derived from a KP GEMM. To avoid adaptation to chronic Opa1 deletion, we used mixed-population cells within a week of viral transduction and observed robust depletion of Opa1 expression (Figure 1A). To assess the effects of acutely inhibiting Opa1 on individual cell growth and survival, we performed colony formation assays and found Opa1 depletion significantly inhibits LUAD colony formation (Figure 1B). We also confirmed this effect in mixed-population human *KRAS*-mutant A549 LUAD cells (Figures 1C and 1D). Next, we pharmacologically inhibited Opa1 using MYLS22^30^ and validated inhibition of mitochondrial fusion in KPY40, A549, and mouse embryonic fibroblasts by examining mitochondrial morphology. DMSO-treated cells demonstrate mixed mitochondrial morphology, whereas MYLS22-treated cells demonstrate fragmented morphology (Figures 1E, S1A, and S1B) similar to that observed by others upon Opa1 deletion.^7,10^ To assess whether pharmacological inhibition of Opa1 affects colony formation, we treated KPY40 cells with DMSO or MYLS22. MYLS22-treated cells demonstrate decreased colony formation at doses of 25 μM and 50 μM (Figure 1F). Together, these data demonstrate that genetic or pharmacologic inhibition of Opa1 decreases KRas-mutant LUAD colony formation.

To determine whether the effects of Opa1 inhibition are mediated by unopposed mitochondrial fission, we examined KPY40 colony formation following Drp1 deletion, Opa1 deletion, or both. We again used CRISPR to inactivate Drp1 first and Opa1 second. We observed complete Drp1 deletion and robust depletion of Opa1 in mixed-population cells but no effect on mitochondrial markers SDHA and VDAC, indicating that acute deletion of Drp1 and/or Opa1 does not alter mitochondrial abundance (Figure 1G). Notably, inactivation of mitochondrial fission by Drp1 deletion does not affect colony formation in these cells (Figures 1H and 1I). This was surprising given that Drp1 deletion inhibits KP PDAC growth. Further, inactivation of mitochondrial fission completely rescues the effects of Opa1 knockout on colony formation. These results indicate that mitochondrial fission mediates the effects of Opa1 knockout on *in vitro* LUAD colony formation and suggest a functional link between Drp1 and Opa1.

### Deletion of Opa1, but not Drp1, inhibits KP LUAD development *in vivo*

**Figure 2.**
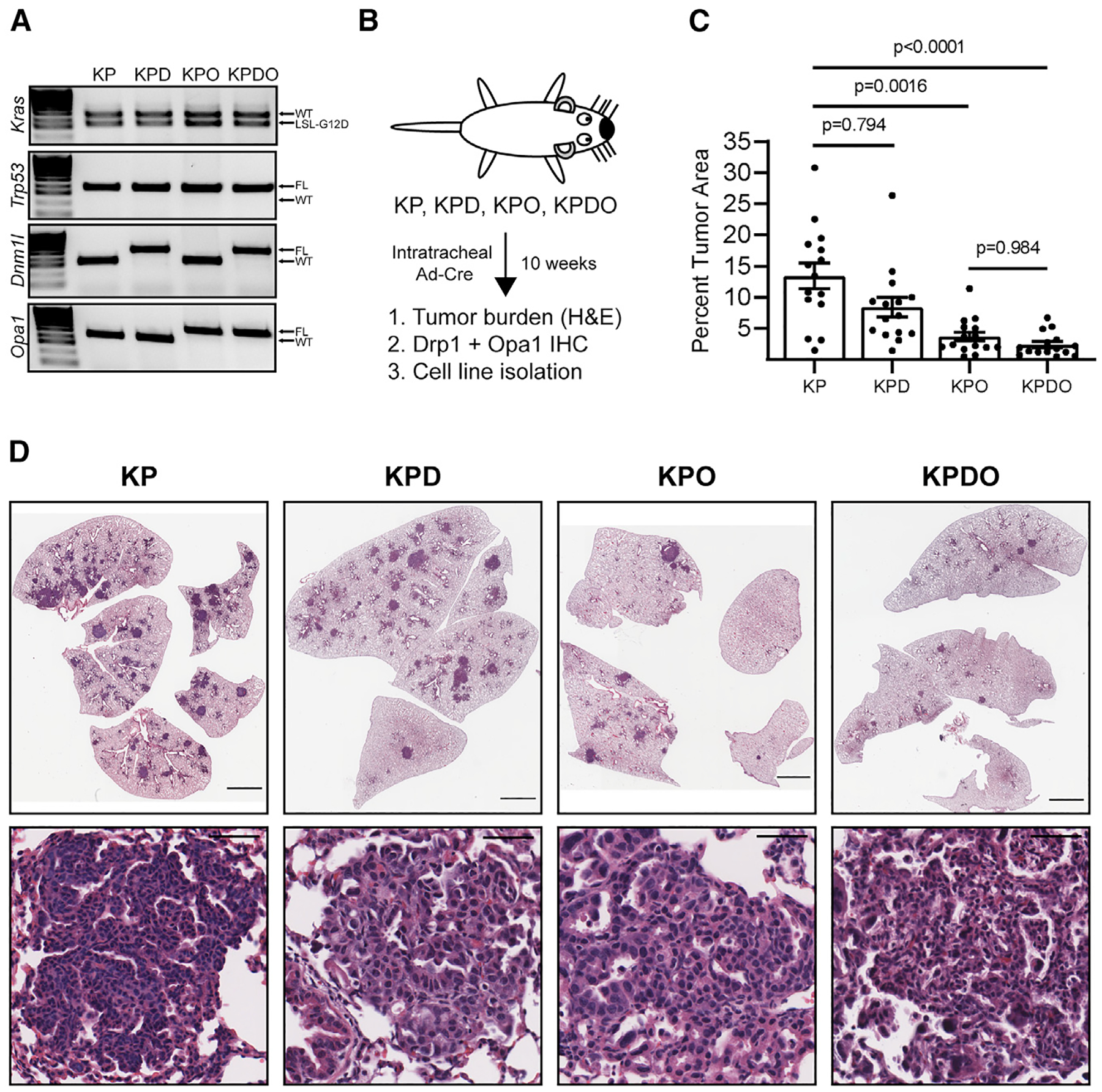
Deletion of Opa1, but not Drp1, inhibits KP LUAD development *in vivo* (A) PCR of *kras, Trp53, Dnm1l*, and *Opa1* alleles in GEMM-enrolled mice. (B) GEMM schematic and endpoints. (C) Tumor burden in individual GEMM mice. n = 15 mice per genotype. Mean ± SD. Kruskal-Wallis test + Dunn’s multiple comparisons test. (D) Representative H&E-stained lungs and tumors in indicated genotypes. Scale represents 2000 μm (lung), 50 μm (tumors).

To test whether deletion of Drp1, Opa1, or both affects spontaneous KP LUAD development *in vivo*, we generated KP mice expressing wild-type Drp1 (*Dnm1l*) and Opa1 (*Opa1*), or with homozygous floxed alleles of *Dnm1l* (KPD), *Opa1* (KPO), or both (KPDO) (Figure 2A). We initiated tumor formation and mitochondrial dynamics gene deletion by intratracheal administration of adenovirus-Cre (AdCre) and allowed tumors to develop for 10 weeks (Figure 2B). Contrary to what is observed in KRas-driven PDAC,^14^ LUAD tumor burden in KPD mice is not significantly different from that of KP (Figure 2C); however, tumor burden in KPO mice is significantly less than in KP. Surprisingly, simultaneous deletion of Opa1 and Drp1 does not rescue tumor development. H&E-stained lung sections demonstrate that all mice develop tumors with similar histological morphology (Figure 2D). Together, these data suggest that Drp1 is dispensable, but Opa1 is required, for KP LUAD development, and that deletion of Drp1 is insufficient to rescue Opa1 deletion-mediated decrease in tumor development *in vivo*.

Recombination of floxed alleles by AdCre in the KP LUAD model is incompletely efficient.^31^ As a result, unrecombined alleles of genes that play a pro-tumorigenic role can be selected for during tumor development. The degree to which developed tumors retain floxed alleles provides insight to whether their gene products are required for tumor development. For example, there is a strong selective pressure to retain Drp1 expression in a PDAC GEMM with genetics identical to the LUAD model used here.^14^

**Figure 3.**
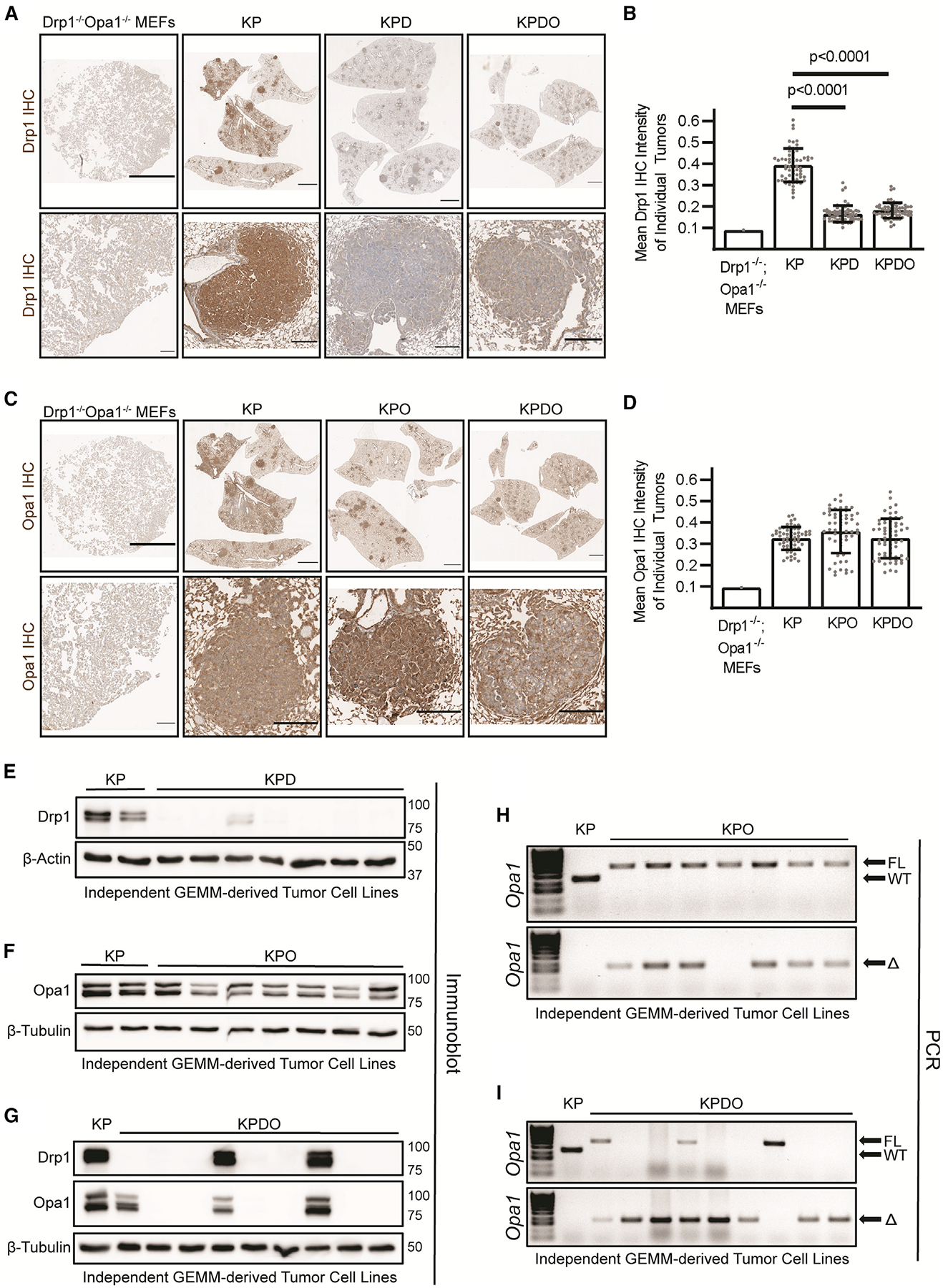
Opa1, but not Drp1, is required for KP LUAD development (A) Representative Drp1 IHC on whole lung and tumors in indicated genotypes. KPDO mouse embryonic fibroblast (MEFs) are negative staining control. Scale represents 2,000 μm (lung), 200 μm (tumors). (B) Quantification of mean Drp1 DAB (3,3′-Diaminobenzidine) intensity of individual tumors in indicated genotypes. n = 60 tumors per genotype. Kruskal-Wallis test + Dunn’s multiple comparisons test. (C) Same as (A), but Opa1 IHC. (D) Quantification of mean Opa1 DAB intensity of individual tumors in indicated genotypes. n = 60 tumors per genotype. (E) Immunoblot of Drp1 in two KP and seven KPD independently derived tumor cell lines. (F) Immunoblot of Opa1 in two KP and seven KPO independently derived tumor cell lines. (G) Immunoblot of Drp1 and Opa1 in one KP and nine KPDO independently derived tumor cell lines. (H) PCR of *Opa1*^*FL*^ and *Opa1*^*WT*^ alleles (top) and *Opa1*^*Δ*^ (recombined, bottom) in one KP and seven independently derived KPO tumor cell lines. (I) Same as (H), but in one KP and nine independently derived KPDO tumor cell lines.

We took two approaches to determine whether individual tumors that form in KPD, KPO, and KPDO mice completely recombine mitochondrial dynamics alleles. First, we performed immunohistochemistry (IHC) on lung sections using antibodies targeting Drp1 or Opa1. Second, we isolated independent tumor cell lines from mice to assess the presence of Drp1 and Opa1 by immunoblot and PCR. Complete recombination of floxed *Kras* and *Trp53* alleles confirms pure tumor cell lines lacking stromal contamination (Figure S2A). KPD and KPDO mice exhibit decreased Drp1 IHC intensity in individual tumors compared with KP (Figures 3A and 3B), while KPO and KPDO tumors exhibit no significant decrease in Opa1 levels (Figures 3C and 3D). Consistent with this, six of seven individually derived KPD tumor cell lines exhibit no detectable Drp1 by immunoblot (Figure 3E), while all seven independently derived KPO tumor cell lines retain lower, but detectable, expression of Opa1 (Figure 3F). Interestingly, six of nine KPDO cell lines demonstrate complete deletion of Opa1 *in vivo*, but only in cells that exhibit complete deletion of Drp1 (Figure 3G). Three additional KPDO cell lines retain expression of Opa1 with or without retained Drp1 expression. Consistent with these data, we detect a single floxed *Opa1* allele by PCR in six of seven KPO cell lines and two floxed alleles in the other (Figure 3H), indicating that a single copy of *Opa1* is sufficient for tumor development. In KPDO mice, six of nine tumor lines harbor homozygous recombined *Opa1*^*Δ*^ alleles, while the others retain one or both *Opa1*^*FL*^ alleles (Figure 3I). Together, these data suggest that mitochondrial fission is dispensable for KP LUAD development *in vivo*, and that Opa1 is essential in tumor cells with intact mitochondrial fission. Further, inactivation of mitochondrial fission permits Opa1-null tumor cell line isolation but not *in vivo* tumor development.

### Opa1 is required to maintain mitochondrial NAD^+^ regeneration

**Figure 4.**
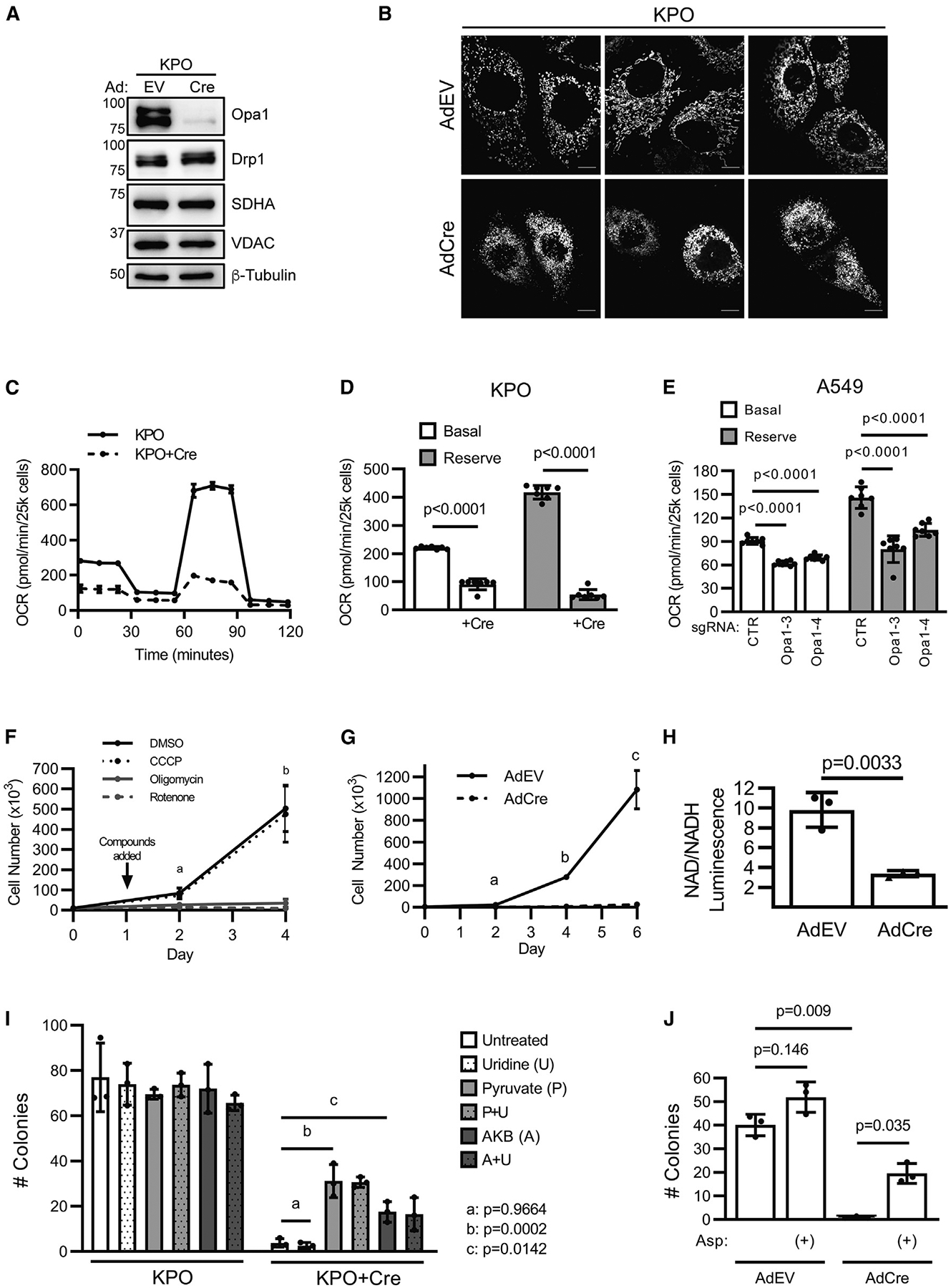
Opa1 is required to maintain mitochondrial NAD^+^ regeneration (A) Immunoblot of Opa1, Drp1, SDHA, and VDAC in AdEV- and AdCre-infected GEMM-derived KPO tumor cells. (B) Mitotracker green-stained AdEV- or AdCre-infected KPO LUAD. Scale represents 10 μm. (C) OCR of uninfected or AdCre-infected KPO. n = 7 wells per cell condition. Mean ± SD. (D) Basal and reserve OCR of uninfected or AdCre-infected KPO cells. n = 7 wells per cell condition. Mean ± SD. Student’s t test. (E) OCR of Opa1 A549 CRISPR cells. n = 7 wells per cell condition. Mean ± SD. One-way ANOVA + Dunnett’s multiple comparisons test. (F) Cell accumulation of Opa1-expressing KPO cells treated with DMSO, oligomycin (10 nM), CCCP (250 nM), or rotenone (250 nM). n = 3 independent experiments. Mean ± SD. a: DMSO vs. oligomycin/rotenone (p < 0.005), DMSO vs. CCCP (p = 0.781), one-way ANOVA + Dunnett’s multiple comparisons test. b: DMSO vs. oligomycin/rotenone (p < 0.05), DMSO vs. CCCP (p = 0.989), Welch ANOVA + Dunnett’s T3 multiple comparisons test. (G) Cell accumulation of AdEV- or AdCre-infected KPO cells. a: p = 0.0041, b: p = 0.0018, c: p = 0.0091. Welch’s t test. (H) NAD^+^/NADH in AdEV- or AdCre-infected KPO cells. n = 3 independent experiments. Mean ± SD. Student’s t test. (I) Colony formation in uninfected or AdCre-infected KPO cells without treatment or treated with uridine (0.1 mg/mL), pyruvate (1 mM), alphaketobutyrate (AKB, 1 mM), or in combination. n = 3 independent experiments. Mean ± SD. One-way ANOVA + Dunnett’s multiple comparisons test. (J) Colony formation in AdEV- or AdCre-infected KPO cells with or without aspartate (20 mM). Welch’s ANOVA + Dunnett’s T3 multiple comparisons test.

Opa1 regulates apoptosis, OXPHOS, and mitochondrial fusion. To understand what drives reduced tumor burden in KPO mice, we explored the consequences of Opa1 deletion *in vitro* using a KPO tumor cell line that retained both *Opa1*^*FL*^ alleles and thus is initially functionally KP. Infection of this cell line *in vitro* with AdCre, but not empty vector adenovirus (AdEV), deletes Opa1 (Figure 4A), whereas AdCre infection of a KP cell line has no effect on Opa1 expression (Figure S3A). Opa1 protein in KPO cells is almost completely undetectable 3 days after AdCre infection, which allows determination of the acute effects of Opa1 deletion. Expression of Drp1, VDAC, and SDHA are not changed by Opa1 deletion, indicating mitochondrial fission remains active and mitochondrial abundance is unaffected (Figure 4A).

As deletion of Opa1 has previously been reported to broadly sensitize cells to apoptosis,^9,10^ we first assessed whether Opa1 deletion decreases cell viability by treating cells with established apoptosis inducers cisplatin or etoposide. Surprisingly, Opa1 deletion slightly increases relative viability of cisplatin- or etoposide-treated cells and does not alter the impact of these agents on cell accumulation (Figures S3B and S3C). Additionally, Opa1 deletion does not increase cisplatinor etoposide-induced poly (ADP-ribose) polymerase cleavage (Figure S3D).

We next assessed whether Opa1 deletion inhibits tumor cell growth by inhibition of mitochondrial fusion. First, we examined mitochondrial morphology in AdEV- or AdCre-treated KPO cells and confirmed that Opa1 deletion causes mitochondrial hyperfragmentation (Figure 4B) as others have described.^7,10^ To determine if inhibition of fusion is sufficient to account for the effects of Opa1 deletion, we prevented fusion independently of Opa1 through CRISPR-mediated deletion of both Mfn1 and Mfn2. Clonal Mfn1/2-targeted CRISPR cells demonstrate complete Mfn2 deletion and robust Mfn1 depletion compared with clonal control cells (Figure S3E). As expected, Mfn1/2 deletion causes fragmented mitochondrial morphology. However, it does not affect colony formation (Figures S3F and S3G). Together, these data suggest that Opa1 deletion does not inhibit tumor growth by inducing apoptosis or through inhibition of mitochondrial fusion.

Opa1 deletion impairs OXPHOS capacity in mouse fibro-blasts.^5–8^ To test whether LUAD cells are similarly sensitive, we performed Seahorse mitochondrial stress tests and found that acute Opa1 deletion severely impairs basal and reserve oxygen consumption (OCR) in KPO cells (Figures 4C and 4D). This effect is not observed in KP cells and thus not due to AdCre infection itself (Figures S4A and S4B). We observe identical results in A549 cells following CRISPR-mediated Opa1 deletion (Figures 4E and S4C).

ETC dysfunction following Opa1 deletion decreases ATP synthesis in mouse fibroblasts^5,6,32^; however, recent evidence suggests that cancer cells primarily fulfill ATP requirements through glycolysis and instead require the ETC to couple electron flux to the oxidation of the pyrimidine precursor dihydroorotate (DHO) to orotate and NADH to NAD^+^, a cofactor essential for oxidative biosynthesis.^27–29,33,34^ We hypothesized that Opa1 deletion-mediated ETC dysfunction impairs KP LUAD growth by inhibiting the ETC electron flux required for oxidation of DHO and NADH, and that mitochondrial ATP synthesis is dispensable. To determine which ETC functions are required in this model, we treated fusion-fission intact Opa1-expressing KPO cells with DMSO, oligomycin, rotenone, or carbonyl cyanide *m*-chlorophenyl hydrazone (CCCP) (collectively ETCi) and measured cell accumulation. CCCP inactivates mitochondrial ATP synthesis through proton gradient dissipation, as confirmed by tetramethylrhodamine, ethyl ester staining (Figure S4D), but it leaves ETC electron flow intact (Figure S4E). Conversely, rotenone and oligomycin diminish both mitochondrial ATP synthesis and ETC electron flux. Oligomycin and rotenone almost completely inhibit cellular accumulation compared with DMSO, while CCCP has no effect, indicating that ETC-mediated oxidation of electron carriers, but not mitochondrial ATP synthesis, is required for KP LUAD growth (Figure 4F). Notably, under seeding conditions almost identical to those used in ETCi experiments, Opa1 deletion phenocopies oligomycin/rotenone treatment, consistent with the effects of its deletion being due to inhibition of ETC function (Figure 4G).

To assess whether Opa1 deletion affects NAD^+^ metabolism, we measured NAD^+^/NADH and found that Opa1 deletion severely reduces this ratio, indicating dysfunction of NADH oxidation (Figure 4H). Impairment of ETC electron flux causes auxotrophy for pyruvate, which supports cytoplasmic NAD^+^ regeneration through lactate dehydrogenase (LDH), and for uridine, which restores pyrimidine synthesis when DHO is unable to be oxidized.^27,29,35^ To test whether Opa1 deletion inhibits KP LUAD colony formation by inhibition of pyrimidine synthesis, NAD^+^ regeneration, or both, we treated cells with uridine alone and in combination with pyruvate and the alternative NAD^+^-regenerating LDH substrate, alphaketobutyrate (AKB). Colony formation of Opa1-expressing cells is unaffected by treatment with uridine, pyruvate, or AKB (Figures 4I and S4F), indicating these cells meet metabolic demands for NAD^+^ and uridine. Notably, pyruvate and AKB increase Opa1-null colony formation, whereas uridine has no effect either alone or with pyruvate or AKB (Figure 4I). These data suggest that Opa1 deletion impairs colony formation by inhibiting NAD^+^ regeneration but not pyrimidine synthesis. To determine whether Opa1 deletion sensitizes cells to inhibition of cytoplasmic NAD^+^ regeneration, we measured viability of tumor cells treated with DMSO, the lactate dehydrogenase inhibitor GNE-140,^36^ or the PDK inhibitor AZD7545 that activates PDH and drives pyruvate into mitochondria and away from lactate synthesis. We found Opa1-null cells demonstrate decreased viability compared with Opa1-expressing cells when treated with GNE-140 or AZD7545 (Figure S4G) suggesting that Opa1 deletion induces increased reliance on cytoplasmic NAD^+^ regeneration. Recent reports demonstrate that the critical and distal effect of inhibiting ETC-mediated NAD^+^ regeneration is the inhibition of aspartate synthesis required for protein, RNA, and DNA synthesis.^27,29,34,37,38^ Consistent with this, aspartate supplementation significantly increases colony formation of Opa1-null LUAD (Figure 4J). Together, these data indicate that Opa1 deletion inhibits KP LUAD by restricting ETC-mediated NAD^+^ regeneration required for oxidative biosynthesis of metabolites including aspartate.

### Drp1 activity drives Opa1 deletion-mediated ETC dysfunction

**Figure 5.**
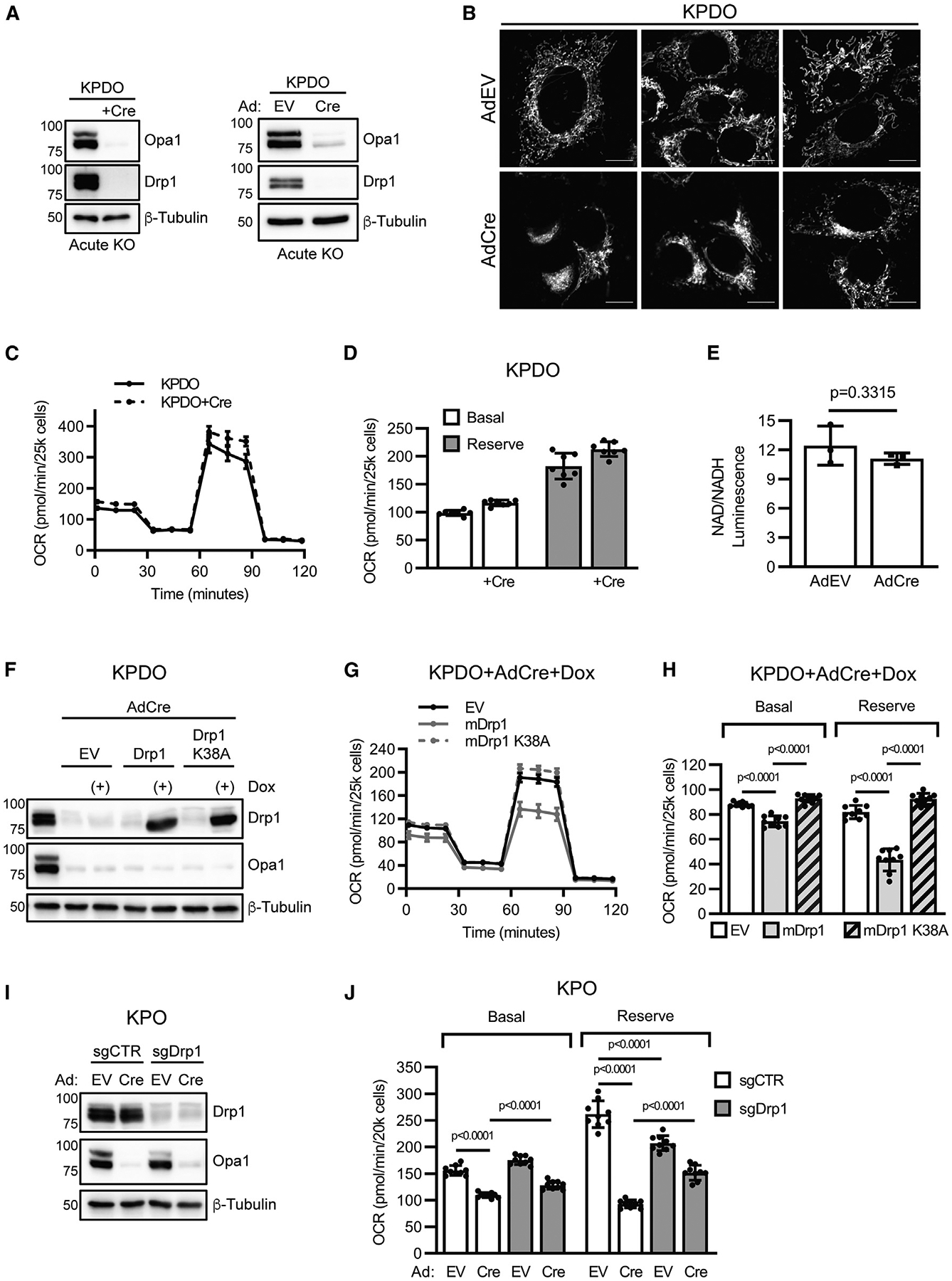
Drp1 activity drives Opa1 deletion-mediated ETC dysfunction (A) Immunoblot of Drp1 and Opa1 in untreated and AdCre-infected KPDO cells (left) and AdEV- or AdCre-infected KPDO (right). (B) Mitotracker green-stained AdEV- or AdCre-infected KPDO cells. Scale represents 10 μm. (C) OCR of untreated or AdCre-infected KPDO cells. n = 7 wells per cell condition. Mean ± SD. (D) Basal and reserve OCR of untreated or AdCre-infected KPDO cells. n = 7 wells per condition. Mean ± SD. (E) NAD^+^/NADH in AdEV- or AdCre-infected KPDO cells. n = 3 independent experiments. Mean ± SD. Student’s t test. (F) Immunoblot of Drp1 and Opa1 in uninfected or AdCre-infected KPDO cells with doxycycline-inducible empty vector (EV), wild-type mouse Drp1 (mDrp1), or GTPase-inactive fission-defective mDrp1^K38A^. (G) OCR of AdCre-infected and doxycycline-induced KPDO cells. n = 9 wells per cell condition. Mean ± SD. (H) Basal and reserve OCR of AdCre-infected and doxycycline-induced KPDO cells. n = 9 wells per condition. Mean ± SD. One-way ANOVA + Sidak’s multiple comparisons test. (I) Immunoblot of Drp1 and Opa1 in mixed-population AdEV- or AdCre-infected control (sgCTR) and sgDrp1 KPO CRISPR cells. (J) Basal and reserve OCR of mixed-population AdEV- or AdCre-infected KPO sgCTR or sgDrp1 CRISPR cells. n = 9 wells per condition. Mean ± SD. One-way ANOVA + Sidak’s multiple comparisons test.

The effect of Opa1 deletion on KP LUAD colony formation is Drp1 dependent (Figures 1H and 1I). Although Drp1 deletion does not rescue *in vivo* tumor growth following Opa1 deletion, we isolated multiple KPDO tumor cell lines with complete *in vivo* deletion of both Opa1 and Drp1, but not a single KPO cell line that completely deleted Opa1 *in vivo*. This suggests an advantage of inactivating fission in the context of Opa1 deletion. To test whether co-deletion of Drp1 and Opa1 in KP LUAD affects ETC function and NAD^+^ regeneration, we utilized a KPDO cell line that retained homozygous *Dnm1l*^*FL*^ and *Opa1*^*FL*^ alleles and thus is initially functionally KP. AdCre infection of this cell line deletes Drp1 and Opa1 within 3 days of infection (Figure 5A). AdEV- and AdCre-treated cells both demonstrate mixed mitochondrial morphology, and while some AdCre cells demonstrate perinuclear mitochondrial aggregation (Figure 5B), they do not demonstrate the hyperfragmented morphology observed in AdCre-infected KPO cells (Figure 4B). Notably, basal and reserve OCR are unaffected by simultaneous deletion of Drp1 and Opa1 (Figures 5C and 5D). Further, NAD^+^/NADH ratio is unaffected by co-deletion of Drp1 and Opa1 (Figure 5E), confirming that the effects of Opa1 deletion on ETC function and NAD^+^ regeneration require Drp1.

Next, we assessed whether ETC function of KPDO LUAD cells with simultaneous Drp1 and Opa1 deletion is sensitive to reintroduction of wild-type mouse mDrp1^WT^ or the GTPase-inactive and fission-defective mutant mDrp1^K38A^. We introduced doxy-cycline-inducible empty vector (EV), mDrp1^WT^, or mDrp1^K38A^ into KPDO+AdCre cells (Figure 5F) and imaged mitochondria (Figure S5A). EV-expressing KPDO+AdCre cells demonstrate mixed mitochondrial morphology with perinuclear aggregates, as observed in KPDO+AdCre cells, whereas mDrp1^WT^-expressing cells demonstrate mitochondrial hyperfragmentation similar to KPO+AdCre cells. mDrp1^K38A^-expressing KPDO+AdCre cells demonstrate mixed morphology and perinuclear aggregates similar to EV-expressing cells, suggesting that fission is inactive in these cells. Expression of mDrp1^WT^ decreases basal and reserve OCR in KPDO+AdCre cells, whereas expression of mDrp1^K38A^ does not (Figures 5G and 5H). As an additional approach, we constitutively expressed luciferase, mDrp1^WT^, or mDrp1^K38A^ in a separate KPDO cell line that retained Opa1 but deleted Drp1 *in vivo* and found that expression of mDrp1^WT^, but not EV or mDrp1^K38A^, inhibits colony formation (Figure S5B). As a third approach, we generated control (sgCTR) and Drp1-targeted KPO CRISPR cells to establish KP, KPO, KPD, and KPDO conditions upon AdCre infection (Figure 5I). A clonal control cell line demonstrates mixed mitochondrial morphology, whereas two independent sgDrp1 clones demonstrate complete mitochondrial tubulation, confirming loss of mitochondrial fission (Figure S5C). As expected, Opa1 deletion in sgCTR cells significantly decreases basal and reserve OCR, and Drp1 deletion rescues this phenotype (Figures 5J and S5D), although more modestly than in KPDO cells with simultaneous Cre-mediated Opa1 and Drp1 deletion. Additionally, acute Drp1 deletion alone mildly decreases OCR. Collectively, these data suggest that mitochondrial fission mediates the effects of Opa1 deletion on ETC function, NAD^+^ regeneration, and colony formation.

### Drp1 mediates ETC disassembly and dysmorphic cristae following Opa1 knockout

The ETC is embedded in the inner mitochondrial membrane (IMM), and its function is dependent on cristae morphology.^5^ Although Opa1 is a key regulator of cristae remodeling,^7,9,10,39^ its exact function in this context is unclear. The role of Drp1 in cristae remodeling is also unclear, but fission promotes apoptotic cristae disorganization.^4^ We hypothesized that Drp1 disrupts cristae during steady-state mitochondrial dynamics, not only during apoptosis, and that Opa1 reorganizes cristae following fission events.

**Figure 6.**
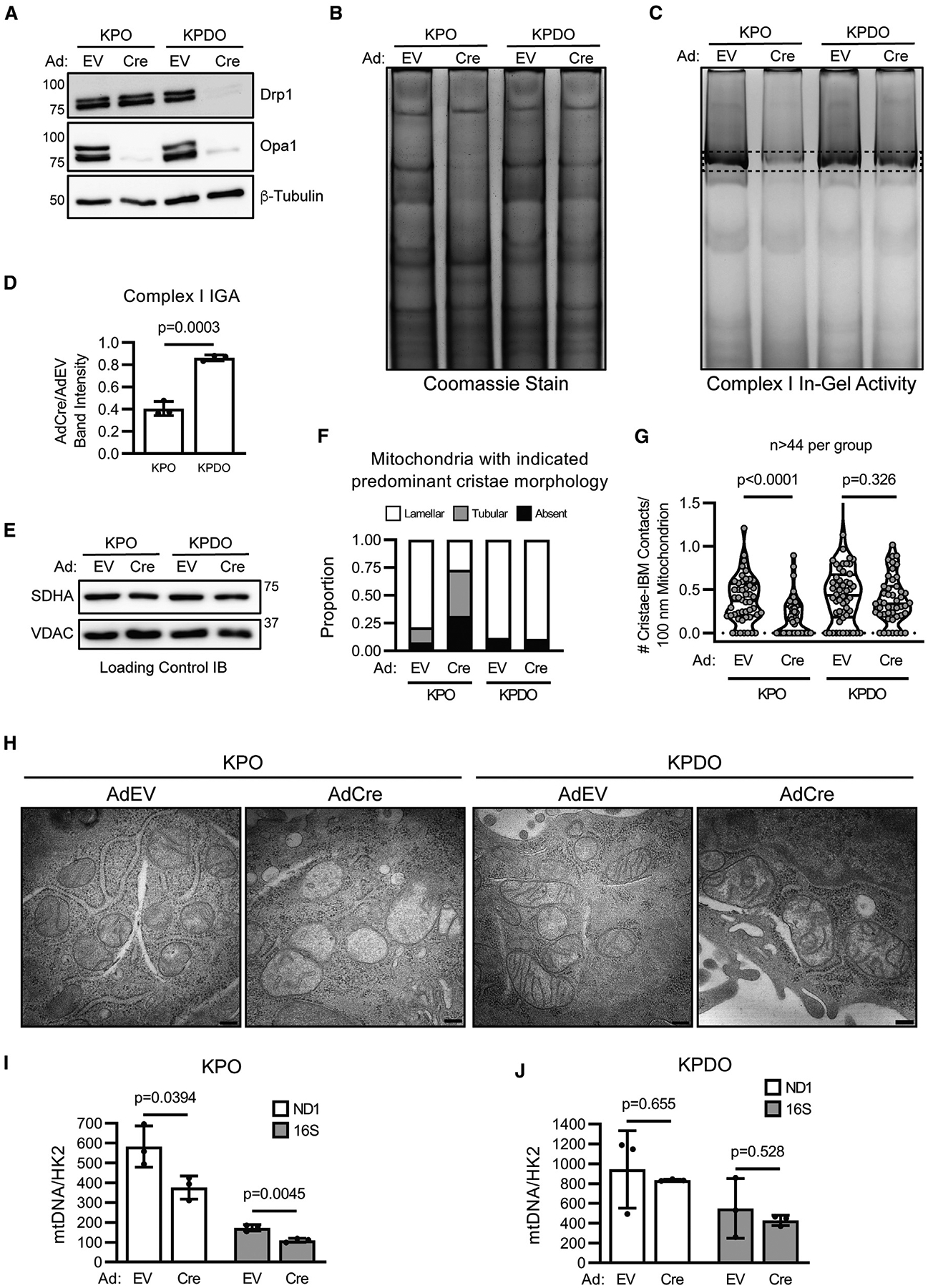
Drp1 mediates ETC disassembly and dysmorphic cristae following Opa1 knockout (A) Immunoblot of Drp1 and Opa1 in AdEV- and AdCre-infected KPO and KPDO cells. (B) Coomassie-stained clear-native PAGE of mitochondrial isolates from AdEV- or AdCre-infected KPO and KPDO cells. n = 3 independent experiments. (C) Clear-native PAGE + complex I in-gel activity (IGA) assay of mitochondrial isolates from AdEV- or AdCre-infected KPO and KPDO cells. n = 3 independent experiments. (D) Quantification of complex I IGA assay intensity. n = 3 independent experiments. Mean ± SD. Student’s t test. (E) Immunoblot of SDHA and VDAC in native PAGE samples. (F) Transmission electron microscopy (TEM) quantitation of dominant cristae morphology (lamellar, tubular, or absent) in individual mitochondria from AdEV- or AdCre-treated KPO and KPDO cells. (G) TEM quantitation of number cristae contacting the inner boundary membrane (IBM) per 100 nm per mitochondrion in AdEV- or AdCre-treated KPO and KPDO cells. n > 44 mitochondria per condition. Violin plot shows quartiles and median. Mann-Whitney test. (H) Representative TEM images in AdEmpty- or AdCre-infected KPO and KPDO cells. Magnification = 30 k. Scale represents 200 nm. See also Figure S1. (I) Quantitative PCR abundance of mitochondrial genes *ND1* and *16S* versus nuclear gene *HK2* in AdEV- or AdCre-treated KPO cells. n = 3 independent experiments. Mean ± SD. Student’s t test. (J) Same as (J), but in KPDO cells.

To assess how Opa1 deletion affects ETC function and cristae homeostasis in the presence and absence of Drp1, we used KPO and KPDO tumor cells that delete retained floxed alleles following *in vitro* AdCre infection (Figure 6A). Since we observed a decrease in NAD^+^/NADH in Opa1-deleted cells expressing Drp1, we assessed assembly of ETC complexes and the activity of ETC complex I, which oxidizes NADH to NAD^+^. Notably, AdCre-infected KPO cells, but not KPDO cells, exhibit decreased abundance of multiple assembled ETC complexes visualized by clear-native PAGE (cnPAGE) analysis of mitochondrial extracts (Figure 6B). Further, Opa1 deletion, but not simultaneous Opa1 and Drp1 deletion, decreases complex I activity (Figures 6C and 6D). Equal loading of mitochondrial isolates was confirmed by immunoblot analysis of SDHA and VDAC (Figure 6E). These data suggest that Drp1-expressing cells require Opa1 to maintain ETC assembly and complex I function, and that cells lacking Drp1 preserve functional ETC complexes in the absence of Opa1.

Opa1 disruption depletes mitochondrial DNA (mtDNA) and disrupts cristae morphology.^7,10,40^ We reasoned that either depletion of mtDNA-encoded ETC subunits or disrupted cristae could affect ETC assembly and function following acute Opa1 deletion. We therefore analyzed cristae structure and mtDNA abundance in AdEV- or AdCre-infected KPO and KPDO cells. Transmission electron microscopy (TEM) revealed that lamellar cristae, in which the cristae membrane contacts the inner boundary membrane (IBM), are abundant in the mitochondria of Opa1-expressing cells. Opa1 deletion decreases the proportion of mitochondria with lamellar cristae and increases the proportion of mitochondria with tubular or no discernable cristae, but this effect is not observed following co-deletion of Opa1 and Drp1 (Figures 6F–6H and S6). We next assessed whether Opa1 deletion affects abundance of mtDNA relative to nuclear DNA by measuring levels of mtDNA genes *ND1* and *16S* and the nuclear gene *HK2* by quantitative PCR. Acute Opa1 deletion reduces abundance of mtDNA to two-thirds that of Opa1-expressing cells, but not if Drp1 is also deleted (Figures 6I and 6J); however, the impact of acute Opa1 deletion on global mtDNA-encoded ETC protein abundance is uncertain, especially on acute gene deletion timescales. These data suggest that at steady state, mitochondrial fission drives dysmorphic cristae, mtDNA loss, and decreased ETC function if unopposed by Opa1.

### Chronic inhibition of mitochondrial dynamics inhibits LUAD ETC function

Our results demonstrate that the effects of acute Opa1 deletion are mediated by unopposed mitochondrial fission; however, that simultaneous Drp1 deletion rescues colony formation, ETC assembly and function, and cristae morphology *in vitro*, but not tumor development *in vivo*, suggests that additional unidentified factors influence the sensitivity of *in vivo* tumor development to mitochondrial dynamics disruption. Two testable possibilities for this discrepancy are the tumor cell environment and the timing of gene deletion.

**Figure 7.**
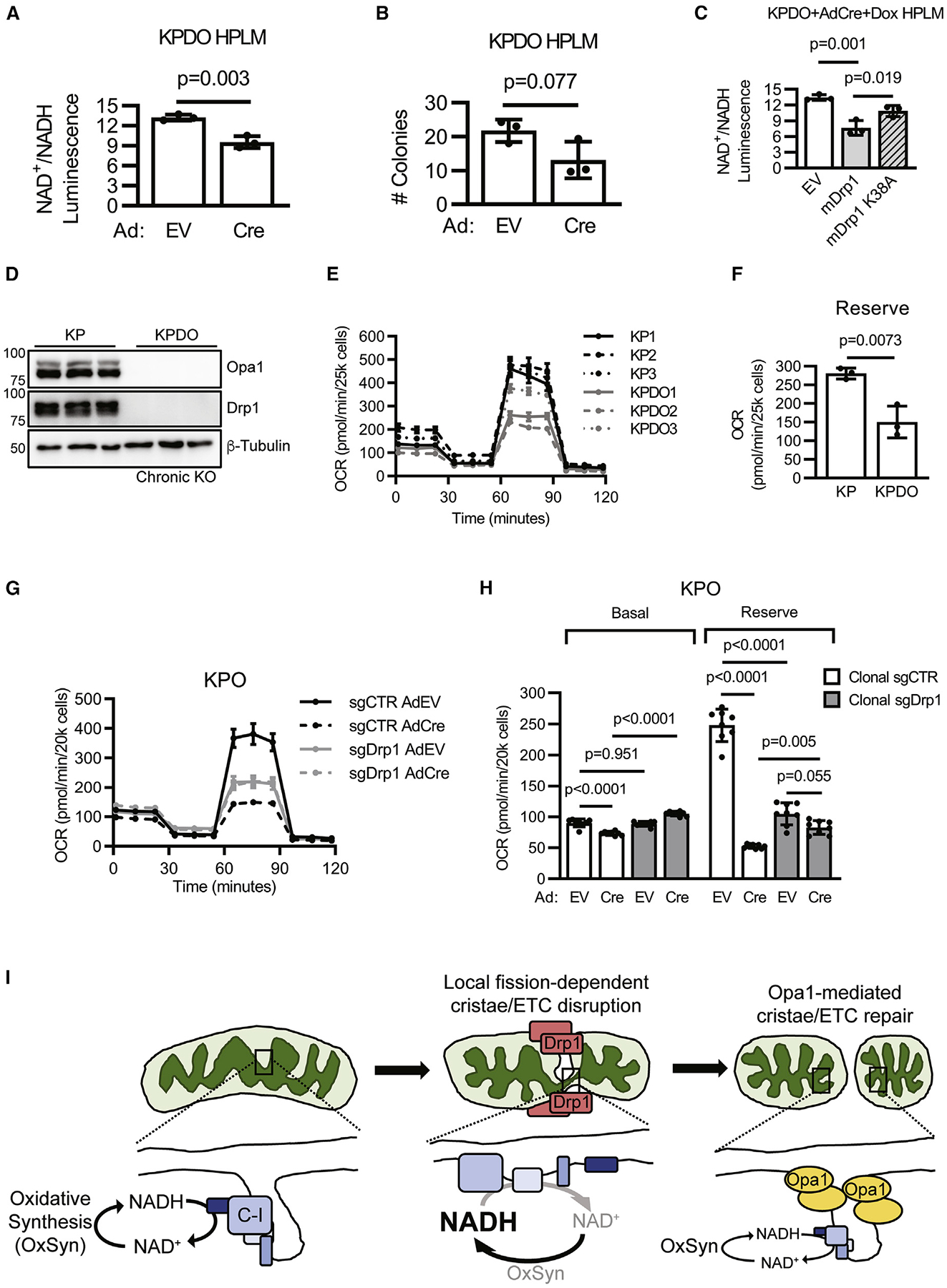
Chronic mitochondrial dynamics gene deletion inhibits LUAD ETC function (A) NAD^+^/NADH in AdEV- or AdCre-infected KPDO cells cultured in HPLM. n = 3 independent experiments. Mean ± SD. Student’s t test. (B) Colony formation in AdEV- or AdCre-infected KPDO cells cultured in HPLM. n = 3 independent experiments. Mean ± SD. Student’s t test. (C) NAD^+^/NADH in AdCre-infected KPDO cells expressing empty vector (EV), mDrp1^WT^, or mDrp1^K38A^ cultured in HPLM. n = 3 independent experiments. Mean ± SD. One-way ANOVA + Sidak’s multiple comparisons test. (D) Immunoblot of Drp1 and Opa1 in KP and KPDO cells. KPDO cells deleted Drp1 and Opa1 *in vivo*. n = 3 independently derived cell lines per genotype. (E) OCR of KP and KPDO cells. n = 3 independent cell lines per genotype. Mean ± SD. (F) Each point = mean reserve OCR of one KP or KPDO cell line. n = 7 wells for each of three independent cell lines per genotype. Mean ± SD. Student’s t test. (G) OCR of clonal control (sgCTR) or clonal Drp1 (sgDrp1) KPO CRISPR cells infected with AdEV or AdCre. n = 8 wells per condition. Mean ± SD. (H) Basal and reserve OCR of clonal control and Drp1 KPO CRISPR acutely infected with AdEV or AdCre. n = 8 wells per condition. Mean ± SD. One-way ANOVA + Sidak’s multiple comparisons test. (I) Model of Drp1 and Opa1 restructuring the inner mitochondrial membrane and their effects on ETC function and NAD^+^ regeneration.

Recent work demonstrated that environment affects tumor metabolism, including that of KRas-mutant LUAD,^41,42^ prompting development of media that resembles human plasma.^43,44^ These media do not perfectly recapitulate the microenvironment of mouse KP LUAD because the tumor microenvironment differs from mouse plasma,^45^ and mouse plasma differs from human plasma^43^; however, they likely model the *in vivo* environment better than high-glucose DMEM. To assess if more-physiologic media affects the rescue of Opa1 deletion by simultaneous Drp1 deletion, we performed colony formation and NAD^+^/NADH assays using KPDO cells (Figure 5A) in human plasma-like medium (HPLM). We found that simultaneous Drp1 and Opa1 deletion in HPLM results in a mild decrease in NAD^+^/NADH (Figure 7A) but does not affect colony formation (Figure 7B). We also found that re-expression of mDrp1^WT^ decreases NAD^+^/NADH compared with EV- or mDrp1^K38A^-expressing cells (Figure 7C), indicating that fission activity promotes a decrease in ETC function in Opa1-depleted cells even in more-physiologic media conditions. These data suggest that differences in extra-cellular metabolite availability likely do not explain the discrepancy between the effects of Drp1 and Opa1 co-deletion on *in vitro* colony formation versus *in vivo* tumor development.

Another distinction between colony formation and the GEMM is the time elapsed between gene deletion and experimental endpoint. We purposefully assessed *in vitro* phenotypes within 1 week of gene deletion to capture acute interactions between Drp1 and Opa1. We assessed GEMM tumor burden at 10 weeks post-initiation to allow enough time for adenocarcinoma-grade lesion development and to maximize assay sensitivity to genetic disruptions that affect tumor development. GEMM tumor burden may therefore reflect both acute and chronic effects of gene deletion due to the 10-fold increase in dynamics disruption time compared with *in vitro* systems. To assess whether chronic disruption of mitochondrial dynamics affects ETC function, we assayed OCR in three independent KP and three independent KPDO cell lines that completely deleted Opa1 and Drp1 *in vivo* (Figure 7D). KPDO cells with *in vivo* Drp1 and Opa1 deletion demonstrate significantly decreased OCR, indicating that ETC function is inhibited under conditions of chronic Drp1 and Opa1 deletion (Figures 7E and 7F). This suggests either that Drp1 deletion leads to loss of ETC function over time, independent of Opa1, or that the ability of Drp1 deletion to restore the effects of Opa1 deletion on ETC function diminish over time. We next generated control and Drp1-targeted KPO CRISPR clones over the course of 6 weeks and assayed OCR after acute AdEV or AdCre infection. We found that chronic Drp1 deletion inhibits OCR in the presence of Opa1 (Figures 7G and 7H), consistent with a role for Drp1-dependent fission in the long-term maintenance of mitochondrial function. Notably, acute Opa1 deletion in chronic Drp1 CRISPR cells has no significant effect on OCR, suggesting that even chronic inactivation of fission mitigates the effects of Opa1 deletion on ETC function. Collectively, these data indicate that chronic Drp1 deletion leads to decreased mitochondrial function but is still protective against the effects of acute Opa1 loss. We note the possibility that chronic loss of both Drp1 and Opa1 is more detrimental to ETC function than chronic loss of Drp1 alone, suggesting that disruptions to cristae that require Opa1-mediated repair can arise over time in the absence of Drp1-dependent fission events. In support of this, continuous fission-independent cristae remodeling has been recently reported using live-cell super resolution imaging.^46,47^

Our findings support a model in which steady-state mitochondrial fusion and fission dynamics constantly restructure both outer and inner mitochondrial membrane morphology (Figure 7I). Mitochondrial fission locally disrupts cristae morphology and ETC assembly and function, including the NAD^+^ regeneration required for oxidative synthesis in highly proliferative tumor cells. Opa1 is required for mitochondrial fusion and to repair cristae and ETC at sites of fission. Thus, the consequences of acute Opa1 deletion in Drp1-expressing cells are profoundly fragmented mitochondrial outer membrane morphology with dysmorphic cristae and reduced ETC function. Importantly, this model predicts that the short-term effects of Opa1 deletion can be prevented by simultaneous inactivation of fission, but that long-term inactivation of fusion-fission cycling causes mitochondrial dysfunction.

## DISCUSSION

This work demonstrates that Opa1 is required *in vitro* and *in vivo* for KRas-mutant LUAD growth and development by promoting the ETC-mediated NAD^+^ regeneration necessary for oxidative biosynthesis. We find the cell growth and metabolic phenotypes that arise from Opa1 knockout are reversible *in vitro* by simultaneous deletion of Drp1 or inactivation of its GTPase catalytic domain. This indicates that mitochondrial fission that is unopposed by Opa1 is catastrophic to mitochondrial function and ultimately cell growth and survival. In stark contrast, Drp1 deletion demonstrates no effect on KP LUAD tumor cell growth *in vitro* or on tumor development *in vivo*. This is surprising given the requirement for Drp1 in a PDAC model with identical KP genetics^14^ and in *BRAF*-mutant melanoma, another MAPK-driven tumor.^13^ This suggests that the consequences of disrupting mitochondrial dynamics are tissue specific.

Opa1-dependent ETC functions are likely tissue specific. For instance, skeletal muscle requires Opa1 for ETC-mediated ATP synthesis,^48^ whereas tumors require ETC-mediated NAD^+^ regeneration and synthesize ATP through glycolysis. Recent work has found that mitochondrial ATP synthesis comes at the expense of rapid NAD^+^ regeneration, as generation of the proton gradient required by mitochondrial ATP synthase slows ETC electron flux and thus NADH oxidation.^28^ It would be interesting to assess whether tumor cells regulate Drp1 and Opa1 to maximize ETC flux and NADH oxidation at the expense of ATP synthesis, potentially through fission-mediated increase in proton leakage to counteract the slowing of ETC electron flux.

Pharmacological Opa1 inhibition may be therapeutically valuable for highly proliferative tumors with substantial ETC-mediated NAD^+^ regeneration requirements and hyperactive mitochondrial fission, such as those with activating mutations in *KRAS* or other MAPK activators. Given KP LUAD can develop with heterozygous deletion of Opa1 and decreased Opa1 protein expression, we suspect that Opa1 activity must be inhibited by more than 50%. It is possible that ETC function in fission-stimulated tumors would be more adversely affected by acute Opa1 inhibition than normal tissues. Supporting this, mouse hepatocytes tolerate complete Opa1 depletion *in vivo*, and Opa1 silencing reverses liver steatosis characterized by megamitochondria.^49^

Drp1 deletion rescues the effects of acute Opa1 deletion on colony formation and ETC function but not *in vivo* tumor development; however, Drp1 deletion also permits isolation of Opa1-null tumor cell lines, suggesting that Opa1 deletion prevents tumor initiation and that simultaneous Drp1 deletion rescues initiation but not progression. Notably, chronic Drp1 deletion leads to decreased ETC function, but it does not inhibit tumor progression in KPD mice. We speculate that tumors with Opa1 and Drp1 deletion undergo initiation, but that ETC dysfunction from combined chronic Drp1 deletion and accumulation of fission-independent cristae disruptions unrepaired by Opa1 inhibit tumor progression. In addition, we cannot rule out other functions of Opa1 that are important *in vivo* but not *in vitro* and that are not impacted by Drp1-dependent fission activity.

Opa1, the mitochondrial contact site and cristae organizing system (MICOS) complex, and ATP synthase dimers regulate cristae morphology^10,50–56^ through mechanisms that remain unclear. Our work suggests that cristae are disrupted by fission and must be repaired to maintain ETC function. The sequence of action of Opa1, MICOS, and ATP synthase dimers in orchestration of cristae structure remains unclear, though recent work suggests Opa1 is epistatic to the core MICOS protein, MIC60.^57^ MIC60 densely populates cristae junctions, whereas Opa1 localizes to cristae membranes and the IBM.^58^ This suggests that Opa1 forms new cristae by directly folding the IMM, while MICOS maintains cristae structure by securing cristae junctions to the outer mitochondrial membrane and inhibiting Opa1 from fusing opposing leaflets of the IMM at cristae junctions. This model is consistent with a requirement for Opa1 only when fission disrupts cristae and predicts that MICOS is required to anchor cristae even in the absence of Drp1. Future work will delineate how fission and MICOS interact to affect cristae structure.

We find that acute Opa1 deletion impacts both cristae morphology and mtDNA content in a Drp1-dependent manner. We cannot rule out the possibility that Drp1 disrupts cristae indirectly through direct loss of mtDNA, which encodes two subunits of ATP synthase. Decreased expression of ATP synthase subunits would decrease assembly and dimer formation, which could disrupt cristae. In this alternative model, Opa1 activity would preserve cristae structure by preventing the fission-induced loss of mtDNA.

Collectively, this work establishes the dependency of LUAD cells on mitochondrial ETC-mediated NAD^+^ regeneration and elucidates how Opa1 opposes Drp1-mediated mitochondrial fission to promote ETC assembly and function through IMM restructuring. This work supports a need for future research that will inform the approach and efficacy of inhibiting mitochondrial dynamics in specific tumor types and the development of more potent and specific inhibitors of dynamics effectors.

### Limitations of this study

Lenti-Cre is more efficient than AdCre at inducing recombination of floxed alleles *in vivo*.^31^ Isolation of GEMM-derived cell lines that retained floxed alleles provides defined tumor genetics and easy genetic manipulation, but incomplete recombination prevents the definitive conclusion that Opa1 is required for Drp1-expressing tumor growth. The impact of this work on human disease requires validation in human cells with heterogeneous genetic backgrounds to understand how alterations in other commonly mutated genes like *TP53* and *STK11* influence this regulation. We recognize that the degree to which Drp1 deletion rescues ETC function following Opa1 deletion is heterogeneous and context dependent. Further exploration of how mitochondrial dynamics regulators interact with each other and tumor genetics, epigenetics, and microenvironment is critical to understand their impact on human disease.

## STAR★METHODS

### RESOURCE AVAILABILITY

#### Lead contact

Further information and requests for resources and reagents should be directed to and will be fulfilled by the lead contact, Dr. David Kashatus (kashatus@virginia.edu).

#### Materials availability

Materials generated in this study including mouse strains, GEMM-derived LUAD cell lines, and vectors will be available upon request of the lead contact.

#### Data and code availability

- All data reported in this paper will be shared by the lead contact upon request.
- This paper does not report original code.
- Any additional information required to reanalyze the data reported in this paper is available from the lead contact upon request.

### EXPERIMENTAL MODEL AND SUBJECT DETAILS

#### KPDO genetically-engineered mouse model (GEMM) of lung adenocarcinoma

This is a fixed endpoint study assessing tumor burden and retention of mitochondrial dynamics gene expression in KP, KPD, KPO, and KPDO mice ten weeks after intratracheal adenovirus-Cre administration. The experimental unit for analysis of tumor burden is the mouse and the sample size is 15 mice per group. The experimental unit for retention of Drp1 and/or Opa1 expression is the individual tumor nodule (IHC, n = 60 per group) or individual GEMM-derived tumor cell line (immunoblot and PCR, sample size variable based on number of independent tumor cell lines generated per mouse genotype). The only inclusion criterion was desired genotype of mice as determined by PCR. All mice that satisfied genotype requirements were enrolled until the sample size within a given group was satisfied. Four KPDO mice past the desired sample size (chronologically the last four) were mistakenly enrolled and infected with adenovirus-Cre. These mice were excluded from tissue harvest for tumor burden analysis to maintain equal group sample sizes but were used to generate GEMM-derived tumor cell lines in the same manner as mice used as intended for tumor burden analysis. No randomization was employed as mice were generated and enrolled based solely on genotype. Mice from both sexes were used in each of the four groups as generated, and the number of each sex is as follows (male, female): KP (9,6), KPD (6,9), KPO (13,2), KPDO (7,8). KP (*Kras*^*LSL-G12D/+*^*; Trp53*^*FL/FL*^) mice were supplied by Dr. Kwon Park. *Opa1*^*FL/FL*^ (O) mice^61^ were provided by Dr. Hiromi Sesaki. KP mice were mated with *Opa1*^*FL/FL*^ and *Dnm1l*^*FL/FL*^ (D)^60^ to generate heterozygous KPO and KPD genotypes. KPD and KPO were mated to generate heterozygous KPDO mice. Breeders for all conditions were descendants from KPDO heterozygotes to maintain recent shared ancestry. KPD, KPO, and KPDO mice were generated by breeding parents heterozygous for floxed *Dnm1l* and/or *Opa1* alleles such that KP controls were generated in the same litters. Male and female mice of desired genotypes were generated from multiple breeder pairs per condition. Primer sequences used for mouse genotyping can be found in Table S1. Intratracheal adeno-virus-Cre infections were performed as previously described.^59^ Briefly, at ten weeks of age, enrollee mice were anesthetized with 250 mg/kg Avertin and intratracheally infected with 2.5 × 10^7^ PFU Adeno-Cre (Baylor Viral Vector Core). Mice were weighed and monitored twice per week for symptoms of disease including weight loss, hyperpnea, and piloerection for ten weeks. At ten weeks post-infection, mice were sacrificed by Avertin anesthetic overdose and exsanguination and lungs were harvested for fixation and/or tumor cell line isolation. At sacrifice, lungs were perfused intratracheally with ice-cold 10% neutral buffered formalin. Mice from which tumor cell lines were derived had the right middle lobe ligated, removed, minced, and seeded into culture medium before perfusion. Perfused lungs were dissociated from the thorax and fixed in tubes containing formalin at 4C. Fixed lungs were paraffin embedded and sectioned to 5 μm thick by the University of Virginia Research Histology Core. Hematoxylin and eosin staining and immunohistochemistry were performed by the University of Virginia Biorepository and Tissue Research Facility. All animal studies were performed in accordance with the University of Virginia Institutional Animal Care and Use Committee. The following parameters were assessed: tumor burden per mouse (measured by tumor surface area versus total lung area on H/E-stained FFPE sections), individual tumor Opa1/Drp1 expression by IHC on FFPE sections, and Opa1/Drp1 expression in GEMM-derived tumor cell lines by immunoblot and PCR. GEMM tumor burden analysis: hematoxylin- and eosin-stained (H&E) lung sections were scanned at high resolution on Aperio ScanScope to generate digital images. Tumor burden was calculated using QuPath software^62^ as percent tumor area (tumor surface area/total lung area). Total lung area, excluding exterior connective tissue, and tumor area were traced using the wand tool. Total lung area per sample was calculated as the sum of the area of each individual lobe per sample. Total tumor area per sample was calculated as the sum of the area of individual tumors present per sample. Representative whole-lung H&E images were pixel-down-sized 10× from original and imported into FIJI. GEMM IHC analysis: IHC lung sections were scanned at high resolution on Aperio ScanScope to generate digital images. IHC DAB intensity was analyzed using QuPath on 20 individual tumors per slide on 3 Drp1-or Opa1-stained slides per genotype assessed (60 total per genotype). All lobes present on slides were sampled. Each tumor had mean DAB intensity measured. Drp1^−/−^;Opa1^−/−^ MEFs were analyzed by removing background first to exclude space between cells and then had mean DAB intensity measured. Statistical analysis of tumor burden and Drp1 IHC intensity was performed by Kruskal-Wallis one-way analysis of variance followed by Dunn’s multiple comparisons test using Prism v7 software. Descriptive statistics of tumor burden results by genotype (mean ± SD): KP (13.43 ± 7.92), KPD (8.37 ± 6.13), KPO (3.68 ± 2.86), KPDO (2.36 ± 1.89). DS, KP, and KK performed anesthesia and intratracheal infection for all mice. DS monitored mice throughout study and performed sacrifice, tissue harvest, and data analysis.

#### KPY40 and KP(D,O,DO) tumor cell line generation

At sacrifice, right middle lobes were aseptically harvested, mechanically minced with a razor blade, trypsin-digested, and plated into 10-cm plates in high-glucose DMEM supplemented with 10% FBS and 1% penicillin/streptomycin. Tumor cells were purified from other cell types initially present in plates by natural selection for cells with oncogenic *Kras* and *Trp53* deletion over successive passages. Purification of tumor cells from non-tumor cells was confirmed by PCR amplification demonstrating recombined *Kras* and *Trp53* alleles, and the absence of *Kras*^*LSL-G12D*^ and *Trp53*^*FL*^ alleles present in non-tumor cells. AdEV- and AdCre-infected cells were used 3–7 days post-infection unless otherwise stated.

#### Cell culture

Human A549 and mouse LUAD cells were cultured in high-glucose DMEM (Life Technologies #11965–092) supplemented with 10% FBS and 1% penicillin/streptomycin (“full DMEM”) at 37C in a 5% CO2 humidified incubator. For indicated uridine-containing media experiments, full DMEM was supplemented with 0.1 mg/mL uridine (Fisher Scientific).

#### Mouse embryonic fibroblast cell line generation

MEFs were prepared as previously described.^63^ Briefly, *Trp53*^*FL/FL*^ (P) mice or *Kras*^*LSL-G12D/+*^; *Trp53*^*FL/FL*^*; Dnm1l*^*FL/FL*^*; Opa1*^*FL/FL*^ (KPDO) mice were mated. At 13.5–14.5 days post-fertilization, pregnant females were euthanized by CO_2_ asphyxiation and cervical dislocation and uterine horns were isolated. Individual embryos were placed in separate dishes and had head and red organs removed with a scalpel and forceps. The remaining tissue was minced and enzymatically digested with trypsin at 37C for 10 min and placed into cell culture in full DMEM. Cell genotype was verified by PCR. Cells were immortalized by *in vitro* Adeno-Cre infection that facilitated homozygous recombination and deletion of the *Trp53* alleles. P MEFs were used for Mitotracker Red staining (Figure S1B). KPDO MEFs were used to validate and optimize Drp1 and Opa1 IHC staining for use on GEMM lung sections (Figures 3A–3D).

### METHOD DETAILS

#### Colony formation assay

100 cells were seeded per well in triplicate in 12-well plates and grown for 1 week in full DMEM. After one week, cells were rinsed once in PBS and fixed in 10% formalin for 20 min at room temperature. Formalin was aspirated and cells were stained with 0.5 mL per well of 0.5% crystal violet solution (0.5 g crystal violet, 20 mL methanol, 80 mL H20) for 10 min at room temperature. Crystal violet solution was removed and cells were rinsed in H_2_0 twice for 10 min on a shaker before drying and imaging. Colony number was quantified using FIJI.

#### CellTiterGlo (CTG) cell viability assay

500 cells were seeded in 50 uL full DMEM in white-walled 96-well plates in technical duplicates or triplicates per independent experiment. The next day, media was changed to 50uL fresh DMEM with indicated compounds. 48 h later, 50 uL CTG reagent (Promega) was added to each well and incubated for 15 min at room temperature before luminescence measurement. Individual wells of drug-treated (non-DMSO) cells were normalized to the mean of the DMSO-treated wells within an individual experiment. Statistical analysis was performed on the mean of normalized values from individual experiments within treatment groups such that the sample size of each treatment group was equal to the number of independent experiments.

#### Seahorse mitochondrial stress test

20,000–25,000 cells were seeded in 80 uL full DMEM per well and placed at room temperature for one hour to adhere before being placed in 37C incubator for another three hours. Assay media was made using Seahorse DMEM supplemented to a final concentration of 25 mM glucose and 4 mM glutamine and pH was adjusted to 7.4 after warming to 37C. Inhibitors were diluted in assay media at the following concentrations: 10× oligomycin (15 uM), 10× CCCP (20 uM), 10× antimycin A/rotenone (10 uM each). Before loading cells, all wells had seeding media aspirated and replenished with 180 uL Seahorse media. Reserve oxygen consumption rate was calculated by subtracting the average resting OCR from the average CCCP-treated OCR. Basal mitochondrial oxygen consumption rate was calculated by subtracting the antimycin A/rotenone-treated OCR from the resting OCR. No normalization was utilized as cells were seeded right before assay start to avoid effects of differential proliferation. All cells within the same cell line were seeded at identical densities.

#### NAD/NADH-Glo assay

50,000 cells were seeded per well in 6-well plates in 2 mL full DMEM. After 24 h, cells were rinsed in PBS, trypsinized, and counted. 5000 cells per condition were assayed per well in white-walled 96-well plates using the Promega NAD/NADH-Glo kit. Cells of independent experiments were seeded and assayed on separate days.

#### Cell accumulation assay

10,000 cells were seeded in 6-well wells in full DMEM in two plates per independent experiment for day 2 and day 4 cell counts. 24 h after seeding, media was replaced to fresh DMEM with DMSO or indicated compounds (oligomycin = 2 × 10^−15^ moles/cell [10 nM], CCCP = 5 × 10^−14^ moles/cell [250 nM], rotenone = 5 × 10^−14^ moles/cell [250 nM]). Cells were trypsinized, harvested, and counted using a hemacytometer on day 2 and day 4. Cells of independent experiments were seeded and assayed on separate days.

#### Mitochondrial isolation

Mitochondrial isolation was performed as previously described.^64^ Cells were cultured in 15 cm plates in full DMEM to full confluence and then trypsinized, harvested, and rinsed once in PBS. The cell pellet was resuspended in 900 uL RSB Hypo Buffer and placed on ice for 15 min to allow cells to swell. Cells were transferred to a 5 mL Dounce homogenizer and homogenized with 20 strokes. Cells were transferred to a new 1.5 mL Eppendorf and 600 uL 2.5X MS homogenization buffer was added and mixed. Cells were centrifuged at 1300 g for 5 min at 4C and the supernatant containing the mitochondrial fraction was transferred to a new tube. The mitochondrial fraction was centrifuged and transferred two more times to remove remaining nuclei or cells. Mitochondria were pelleted by centrifugation at 15000 g for 15 min, rinsed in 1 mL of 1X MS homogenization buffer, and pelleted again at 15000 g for 15 min. Mitochondria were resuspended in buffer (150 mM sodium acetate, 30 mM HEPES, 1 mM EDTA, 12% glycerol (w/v), pH 7.5) at 2 ug protein/uL for further analysis. An aliquot of each lysate was reserved for immunoblot analysis of equal gel loading. Cells of independent experiments were seeded, harvested, and fractionated on separate days.

#### Mitochondrial native *PAGE* and ETC complex I in-gel activity (IGA) assay

High resolution clear native (hrCN) PAGE was performed as previously described.^65^ Briefly, isolated mitochondria were solubilized with 2 ug dodecyl maltoside per ug protein on ice for 30 min. Solubilized mitochondria were centrifuged at 17,000xg for 10 min at 4C. 25 ug solubilized mitochondria was mixed with 1 uL loading buffer (50% glycerol, 0.1% ponceau S) per 10 uL sample. 4–10% acrylamide gradient gels were cast by hand by laying down 1 mL layers at concentrations including and between 4% and 10%. Gels were run at 4C for 1 h at 100V and then 1 h at 200V. After electrophoresis, gels for complex I activity assay were placed in complex I IGA buffer (5 mM Tris pH 7.4, 2.5 mg/mL MTT, 0.1 mg/mL NADH) on a shaker for 15 min at room temperature. After 15 min, buffer was removed, and gel was incubated in 10% acetic acid for 15 min on a shaker and then imaged. Complex I IGA band intensities were quantified using FIJI. After gel electrophoresis, gels for Coomassie stain were placed in 0.025% Coomassie G250 in 10% acetic acid at room temperature for 1 h. Gels were then rinsed overnight in 10% acetic acid in containers with Kim-wipes and imaged.

#### Mitochondrial DNA measurement

Measurement of the ratio of mitochondrial to nuclear DNA was performed as previously described.^66^ Cells were cultured in 10-cm dishes and harvested by trypsinization. Cell pellets were lysed in 600 uL buffer (100 mM NaCl, 10 mM EDTA, 0.5% SDS, 20 mM Tris-HCl pH 7.4, 200 ug/mL proteinase K) at 55C for 3 h. DNA was precipitated with 250 uL 7.5M ammonium acetate and 600 uL 70% isopropanol and centrifuged at 4C for 10 min at 15,000 g. The pellet was rinsed with 70% ethanol and centrifuged again and DNA was resuspended in TE buffer. DNA concentration was measured on a NanoDrop and DNA was diluted to 10 ng/uL for qPCR. qPCR was performed in triplicate for each sample and each primer set in 96-well plates with each well containing 10 uL 2× SYBR green qPCR mix, 8 uL of 10 ng/uL DNA, and 2 uL of 10 uM combined forward and reverse primers. The qPCR program run was 95C (5 min), 45 cycles of 95C (10s), 60C (10s), and 72C (20s), then a melting curve. Copy numbers of mitochondrial:nuclear DNA were calculated by the ΔΔCt method as #copies mtDNA = 2*2^(Ct(HK2)-Ct(ND1 or 16S)). Primer sequences can be found in Table S3. Student’s T test was used for statistical analysis.

#### Transmission electron microscopy

Cultured cells were harvested by trypsinization, rinsed in PBS, and pelleted. Cell pellets were fixed in 1 mL of 4% formaldehyde 0.1M sodium cacodylate at 4C for 1–3 days. Fixative was removed and pellets were rinsed twice in 1 mL 0.1M sodium cacodylate. After the second rinse, cells were placed in osmium tetroxide in 0.1M sodium cacodylate for 45 min at room temperature shaking in the dark. Cell pellets were then rinsed once in 0.1M sodium cacodylate and dehydrated by successive 10-min incubations in 30%, 50%, 70%, 95%, and 100% ethanol. Dehydrated samples were embedded in increasing concentrations (25%, 50%, 75%, 100%) of resin and placed in the oven. Embedded samples were then sectioned and stained with uranyl acetate and lead. Samples were imaged at 30k magnification. Images were analyzed in a randomized and blinded fashion.

#### Mitotracker Green FM staining and imaging

20,000 cells were seeded on glass-bottom dishes (Greiner Bio-One CELLview) dishes in full DMEM. The next day, cells were incubated in 150 nM Mitotracker Green FM in full DMEM for 1 h in a 37C incubator. After incubation, cells were rinsed once in PBS and then placed in live-cell imaging DMEM (25 mM glucose, 4 mM glutamine, 1% FBS, no phenol red). Cells were immediately imaged using a Zeiss LSM900 with Airyscan. Crop area was set to 2× and scan speed was set to 4–9 μs per pixel in Zen Blue software.

#### Plasmid construction and viral transduction cell line generation

##### Drp1 plasmids

Mouse Drp1 was PCR amplified from pcDNA3.1 mDrp1 (Addgene #34706) and cloned into pLenti BlastR or pCW57.1 using InFusion cloning. Drp1 mutants were generated using InFusion cloning.

##### CRISPR

plentiCRISPRv2 was digested with BsmBI and annealed sgRNA oligos were ligated with T4 ligase. For double CRISPR cells, the puromycin resistance transgene from plentiCRISPRv2 was swapped for a neomycin resistance transgene to generate plentiCRISPRv2 NeoR and sgRNAs were cloned in using the same protocol. Lentivirus was generated in 293T cells by calcium phosphate cotransfection with psPAX2, pCMV-VSV-g, and plentiCRISPRv2 vectors. Media was removed from 293T cells 24 and 48 h after transfection, filtered through 0.45 um PES filters, and applied to target cells with polybrene (5 ug/mL). Infected cells were selected in full DMEM with puromycin (3 ug/mL for mouse cells, 1 ug/mL for A549) or neomycin (1 mg/mL mouse and A549). CRISPR cells were used acutely within one week of gene deletion. CRISPR sgRNA sequences can be found in Table S2.

#### Immunoblotting

Cell pellets were lysed on ice in RIPA buffer with protease/phosphatase inhibitor cocktail (Roche). Protein concentration was determined by BioRad Protein Assay (BioRad) and 20 ug protein was resolved by SDS-PAGE in 10% acrylamide gels, transferred to PVDF membrane (Millipore), blocked in 5% fat-free milk, and probed with indicated antibodies.

#### TMRE flow cytometry

500,000 cells were seeded in 10 mL full DMEM in 10-cm plates and adhered overnight. The next day, media was changed to full DMEM with DMSO, oligomycin, or CCCP at equimolar amounts per cell as the cellular accumulation experiment using these same compounds (oligomycin = 2 × 10^−15^ moles/cell [100 nM], CCCP = 5 × 10^−14^ moles/cell [2.5 uM]) and incubated for one hour at 37C. Cells were trypsinized, counted, and 250,000 per treatment were incubated in 500 uL PBS +4 mM glutamine + 25 mM glucose + 100 nM TMRE for 30 min at 37C before analysis by flow cytometry on an Attune NxT flow cytometer. Data were analyzed in FCS Express. TMRE intensity was determined in cells gated solely on the live cell population as determined by SSC x FSC.

### QUANTIFICATION AND STATISTICAL ANALYSIS

Statistical analysis and data presentation were performed using GraphPad Prism v7. All dispersion and precision measures, statistical tests used and significance are indicated in individual figure panels and legends. When applicable, normalization before statistical analysis is described in the methods section for that particular assay (e.g. CTG assays of cell viability). When variation between groups was unambiguously unequal, non-parametric statistical testing was used (e.g. GEMM tumor burden analysis).

## Supplementary Material

1

## Figures and Tables

**Table:** KEY RESOURCES TABLE

REAGENT or RESOURCE	SOURCE	IDENTIFIER
Antibodies		
Mouse monoclonal anti-Opa1 (WB)	BD Biosciences	Cat# 612606; RRID: AB_399888
Rabbit polyclonal anti-Opa1 (IHC)	GeneTex	Cat# 48589; RRID: AB_10623460
Rabbit monoclonal anti-Drp1 (WB + IHC)	Cell Signaling Tech.	Cat# 5391; RRID: AB_11178938
Rabbit monoclonal anti-beta tubulin	Cell Signaling Tech.	Cat# 2128; RRID: AB_823664
Rabbit monoclonal anti-beta Actin	Cell Signaling Tech.	Cat# 8457; RRID: AB_10950489
Rabbit monoclonal anti-PARP	Cell Signaling Tech.	Cat# 9542; RRID: AB_2160739
Rabbit monoclonal anti-SDHA	Cell Signaling Tech.	Cat# 11998; RRID: AB_2750900
Rabbit monoclonal anti-VDAC	Cell Signaling Tech.	Cat# 4866; RRID: AB_2272627
Rabbit polyclonal anti-Mfn1	Proteintech	Cat# 13798; RRID: AB_2266318
Rabbit monoclonal anti-Mfn2	Cell Signaling Tech.	Cat# 9482; RRID: AB_2716838
Bacterial and virus strains		
Ad5-CMV-Cre	Baylor Vector Core	N/A
Ad5-CMV-Empty (EV)	Baylor Vector Core	N/A
Chemicals, peptides, and recombinant proteins		
MYLS22 Opa1 inhibitor	Gift from Luca Scorrano	N/A
Avertin (2-2-2,-tribromoethanol)	Fisher	AAA1870606
Critical commercial assays		
NAD/NADH-Glo	Promega	G9071
Seahorse Mitochondrial Stress Test	Agilent	103015–100
CellTiter-Glo	Promega	G7570
Experimental models: Cell lines		
A549 human lung adenocarcinoma	ATCC	CCL-185
KP, KPD, KPO, KPDO mouse lung adenocarcinoma	This study	N/A
Experimental models: Organisms/strains		
Mouse: KP: *Kras*^*LSL*-*G12DIWT*^*; Trp53*^*FL/FL*^	DuPage et al.^59^	N/A
Mouse: D: *Dnm1l*^*FL/FL*^	Wakabayashi et al.^60^	N/A
Mouse: O: *Opa1*^*FL/FL*^	Zhang et al.^61^	N/A
Oligonucleotides		
Primers and guide RNAs	This study	Please see Tables S1–S3
Recombinant DNA		
plentiCRISPRv2	Addgene	52961
plenti BlastR Luciferase-V5	Addgene	21474
pcDNA3.1 mDrp1	Addgene	34706
pCW57.1	Addgene	41393
psPAX2	Addgene	12260
pCMV-VSV-g	Addgene	8454
Software and algorithms		
Graphpad Prism v8.3	GraphPad	N/A
Fiji	ImageJ	https://imagej.net/software/fiji/downloads
QuPath v0.3.0	Bankhead et al.^62^	https://qupath.github.io/
Aperio ImageScope	Leica Biosystems	N/A
FCS Express	DeNovo Software	N/A
ZEN Black	Zeiss	N/A

## Highlights

- KRas-mutant lung adenocarcinoma requires Opa1, but not Drp1, *in vitro* and *in vivo*
- Acute Opa1 deletion inhibits LUAD by disrupting ETC function, not fusion or apoptosis
- Mitochondrial fission unopposed by Opa1 inhibits ETC assembly and NAD^+^ regeneration
