# Supplementary material for: Opa1 and Drp1 reciprocally regulate cristae morphology, ETC function, and NAD+ regeneration in KRas-mutant lung adenocarcinoma
Source: Cell Rep. Author manuscript; Available in PMC 2023 Jun 14. (PMC10265649; doi:10.1016/j.celrep.2022.111818)
Supplement: 1 [file NIHMS1903394-supplement-1.pdf]

Cell Reports, Volume *41*

Supplemental information

Opa1 and Drp1 reciprocally regulate cristae morphology, ETC function, and NAD^+ regeneration in KRas-mutant lung adenocarcinoma

Dane T. Sessions, Kee-Beom Kim, Jennifer A. Kashatus, Nikolas Churchill, Kwon-Sik Park, Marty W. Mayo, Hiromi Sesaki, and David F. Kashatus

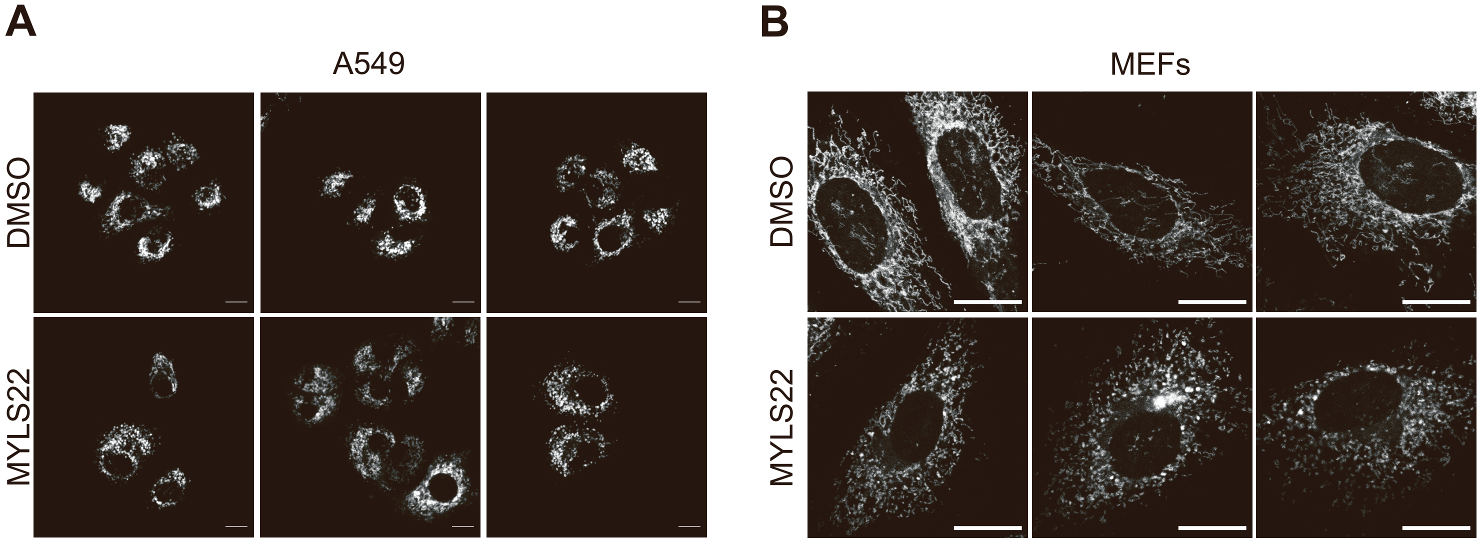

Figure S1: Cells treated with Opa1 inhibitor MYLS22 demonstrate fragmented mitochondrial morphology. Related to Figure 1.

A.Mitotracker Green-stained A549 cells treated with DMSO or 50 µM MYLS22 for 72 hours. Scale = 10 µm.

B.Mitotracker Green-stained MEFs treated with DMSO or 50 µM MYLS22 for 72 hours. Scale = 20 µm.

A

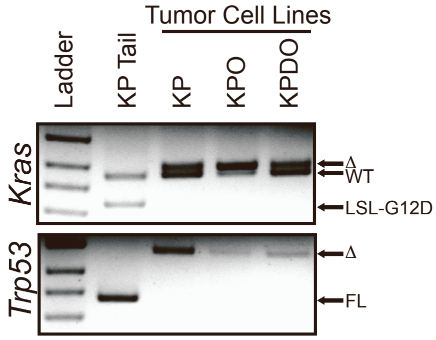

Figure S2: GEMM-derived LUAD cells completely recombine *Kras*$^{LSL\text{-}G12D}$ and *Trp53*^{FL} alleles. Related to Figure 3.

A. *Kras* and *Trp53* PCR genotypes of KP mouse tail and one representative each of KP, KPO, and KPDO GEMM-derived cell lines.

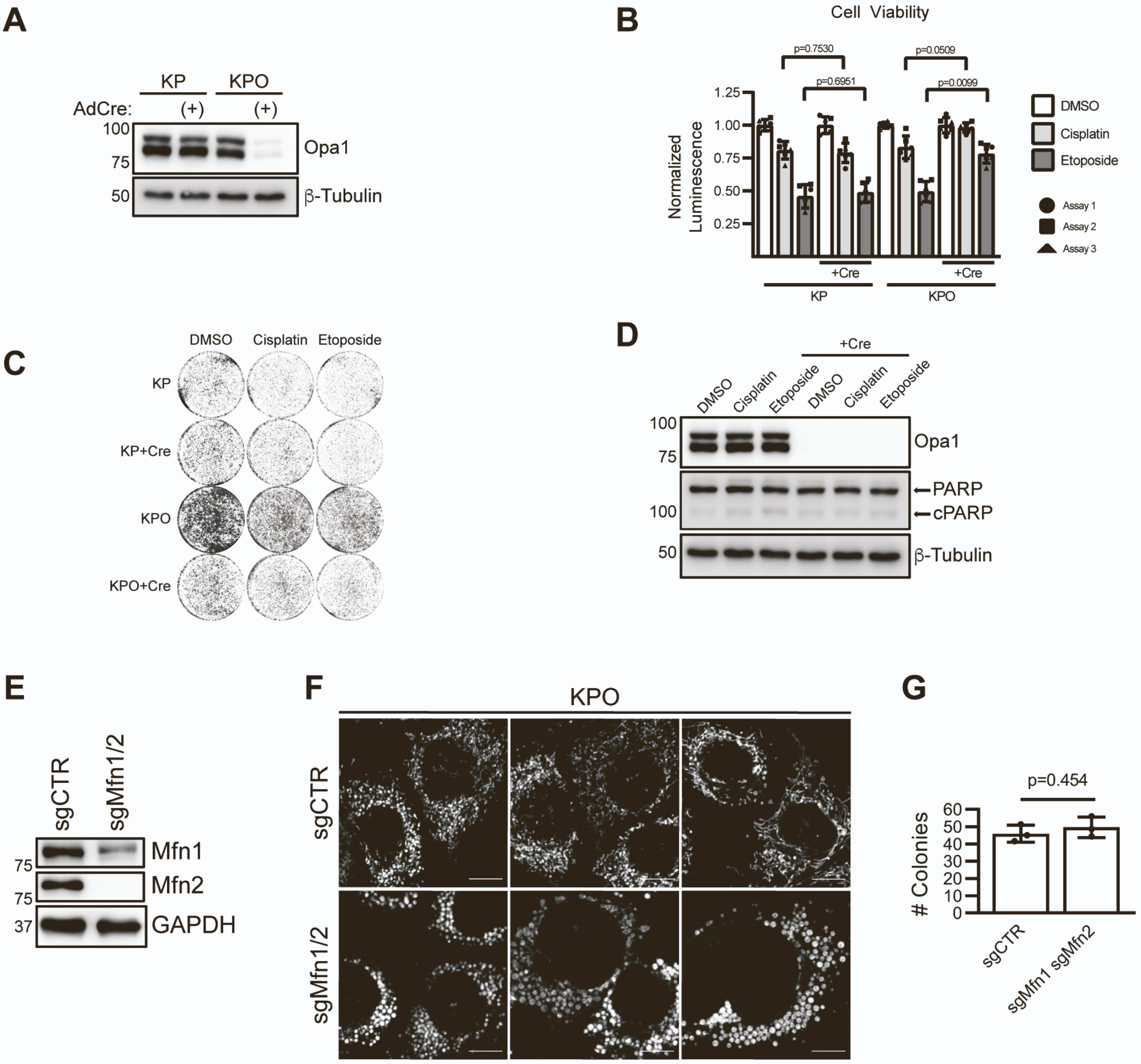

Figure S3: Opa1 deletion does not inhibit LUAD growth by promoting apoptosis or inhibiting mitochondrial fusion. Related to Figure 4.

A.Immunoblot of Opa1 in uninfected or AdCre-infected KP and KPO cells.

B.CellTiter-Glo cell viability of uninfected or AdCre-infected KP and KPO cells treated with DMSO, cisplatin (2 μM), or etoposide (2 μM) for 48 hours. All technical replicates shown. n=3 independent experiments. Mean±SD. Student's T-test.

C.Crystal violet staining of uninfected or AdCre-infected KP and KPO cells treated with DMSO, cisplatin (2 μM), or etoposide (2 μM) for 48 hours.

D.Immunoblot of Opa1 and PARP in uninfected or AdCre-infected KPO cells treated with DMSO, cisplatin (2 μM), or etoposide (2 μM) for 48 hours.

E.Immunoblot of Mfn1 and Mfn2 in clonal control (sgCTR) or clonal Mfn1 and Mfn2 CRISPR (sgMfn1/Mfn2) KPO cells (no Cre).

F.Mitotracker Green-stained control and Mfn1/2 CRISPR KPO cells. Scale = 10 μm.

G.Colony formation of control and Mfn1/2 CRISPR KPO cells. Mean±SD. Student's T-test.

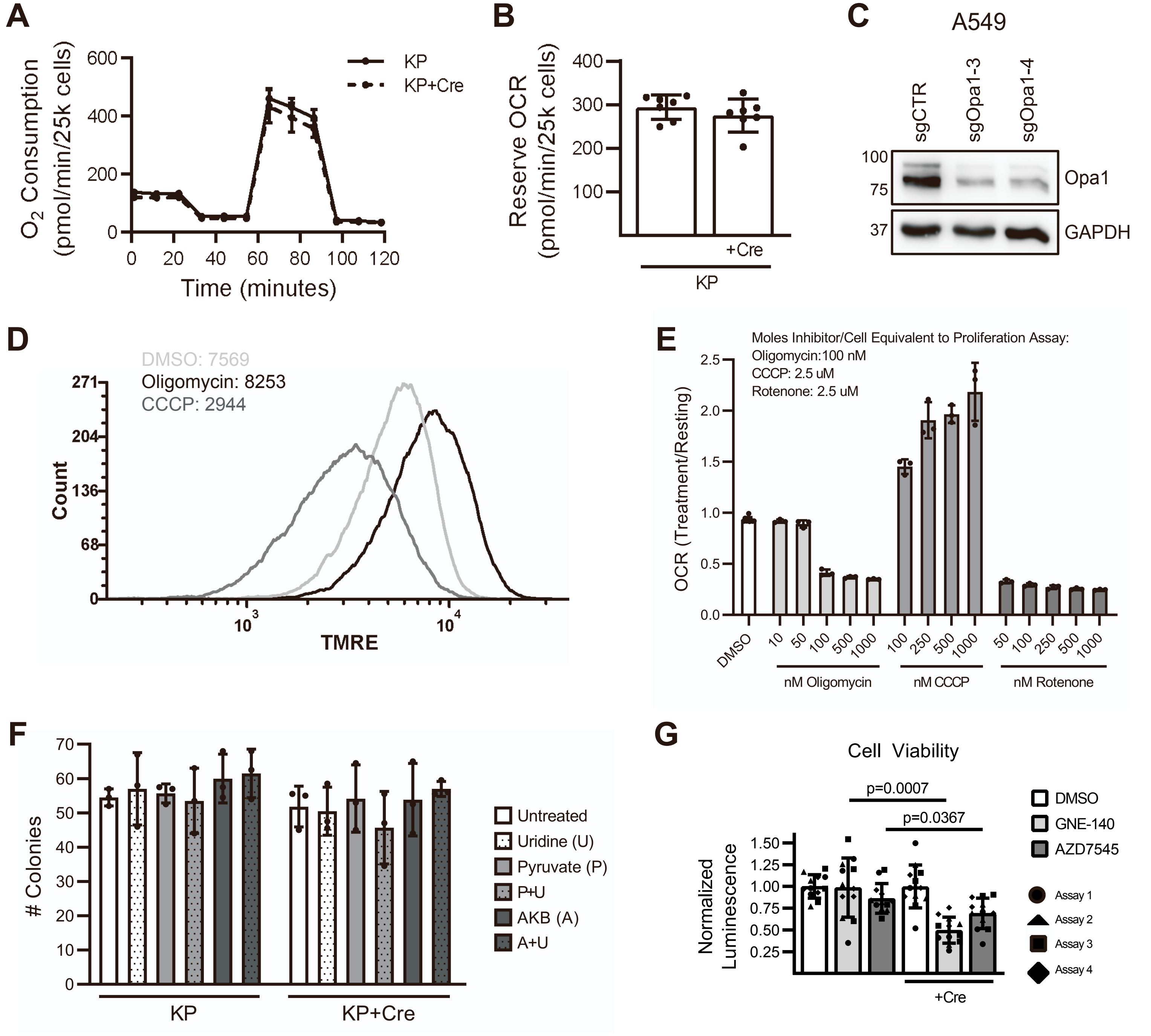

Figure S4: Opa1 deletion inhibits LUAD respiration and sensitizes tumor cells to inhibition of cytoplasmic NAD+ regeneration. Related to Figure 4.

A.OCR of untreated or AdCre-infected KP cells. n=7 wells per cell condition. Mean±SD.

B.Reserve OCR of untreated or AdCre-infected KP cells. n=7 wells per condition. Mean±SD.

C.Immunoblot of Opa1 in control or two independent Opa1 CRISPR A549 cell lines.

D.TMRE flow cytometry of uninfected KPO cells treated with equal moles oligomycin/CCCP per cell as cellular accumulation experiment (Figure 4F). Cells gated only on live cell population (FSCxSSC). Values indicate median fluorescence intensity. n>20,000 cells per group.

E.Treated/resting OCR of uninfected KPO tumor cells with indicated of compounds. n=3 wells per condition. Mean±SD.

F.Colony formation in uninfected or AdCre-infected KP cells without treatment or treated with uridine (0.1 mg/mL), pyruvate (1 mM), alphaketobutyrate (AKB, 1 mM), or in combination. n=3 independent experiments.

G.CellTiter-Glo cell viability in uninfected or AdCre-infected KPO cells treated with DMSO, GNE-140 (5 μM), or AZD7545 (5 μM) for 48 hours. All technical replicates shown. n=4 independent experiments. Mean±SD. Student's T-test.

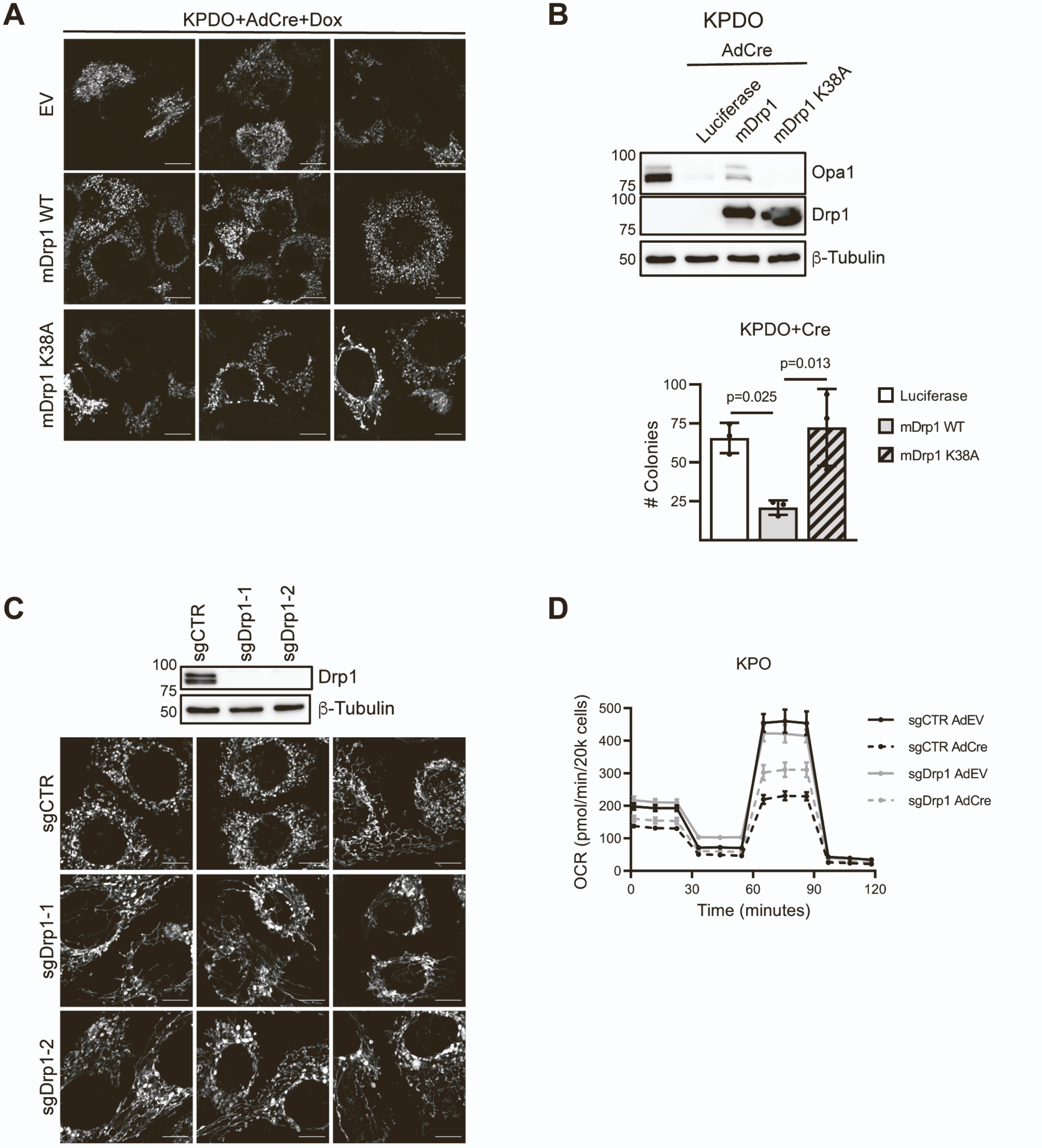

Figure S5: Assessment of LUAD mitochondrial morphology and colony formation under Opa1 and/or Drp1 deletion. Related to Figure 5.

A.Mitotracker Green-stained AdCre-infected KPDO cells with doxycycline-induced empty vector (EV), mDrp1WT, or mDrp1K38A. Scale = 10 μm.

B.Immunoblot of Drp1 and Opa1 and colony formation in KPDO cells that deleted Drp1 but retained Opa1 in vivo and constitutively express luciferase, mDrp1WT, or mDrp1K38A. Mean±SD. One-way ANOVA + Sidak's multiple comparisons test.

C.Immunoblot of Drp1 and Mitotracker Green imaging in one sgCTR and two sgDrp1 KPO CRISPR clonal cell lines. Scale = 10 μm.

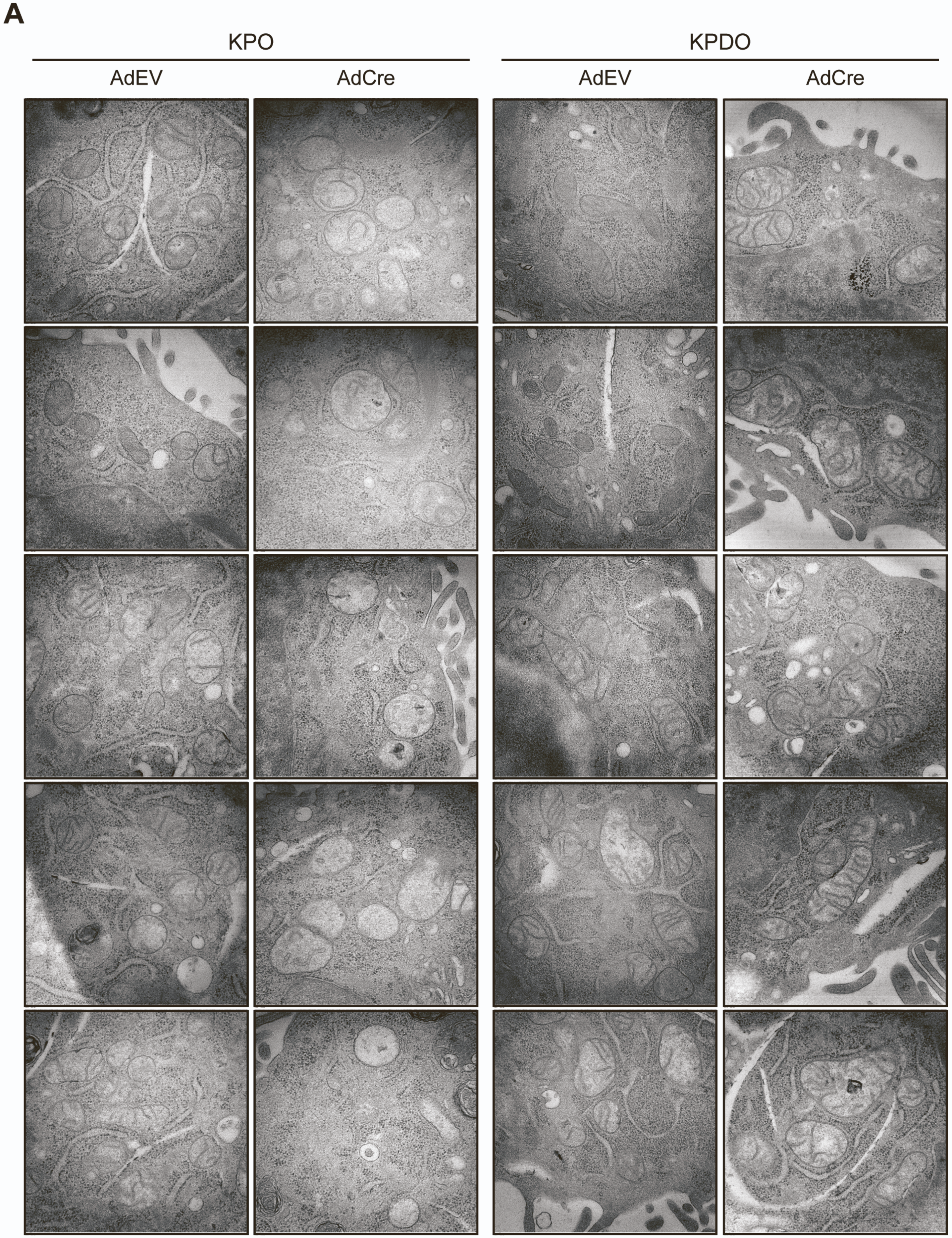

Figure S6: Opa1 deletion impairs mitochondrial cristae morphology in a Drp1-dependent manner. Related to Figure 6.

A.Additional TEM images of cristae morphology in AdEV- or AdCre-infected KPO and KPDO cells. Magnification = 30k. Scale = 200 nm.

Table S1: KPDO Mouse Genotyping Primers (5’-3’)

Kras F1: CTAGCCACCATGGCTTGAGT
Kras F2: ATGTCTTTCCCCAGCACAGT
Kras R: TCCGAATTCAGTGACTACAGATG
Trp53 F: CACAAAAACAGGTTAAACCCAG
Trp53 R: AGCACATAGGAGGCAGAGAC
Dnm1l F: ACCAAAGTAAGGAATAGCTGTTG
Dnm1l R: ATGCGCTGATAATACTATCAACC
Opa1 F: TTAAGACACCCCAAGAGCTTGC
Opa1 R: CCAGCTTAGATCCCATTTGTTGACAG
Opa1 Recombined F: CCATCTGTGTAGTGAACTTACTG
Opa1 Recombined R: TGACGAGGCGTCCGAAGAACGGATCCAAGC

Table S2: CRISPR Guide RNA Sequences (5’-3’)

sgCTR: GTATTACTGATATTGGTGGG
Mouse *Opa1* sg1: GCGCCTGCGAGAGCTCGACA
Mouse *Opa1*/Human *OPA1* sg4: AAATGTAGCCAGTCCAAGCA
Human *OPA1* sg3: TCAGTGGAAAGATATGATAC
Mouse *Dnm1l* sg1: GCACAAATAAAGCAGGACGG
Mouse *Dnm1l* sg2: AATCGTGTTACAATACTCTG
Mouse *Mfn1* sg: GCTCAAGGTTGTAAGTCCGT
Mouse *Mfn2* sg: CCGAGGCAGACGCATCCCAG

Table S3: mtDNA:nDNA Analysis qPCR Primers (5’-3’)

Hk2 F: GCCAGCCTCTCCTGATTTTAGTGT
Hk2 R: GGGAACACAAAAGACCTCTTCTGG
mt-Nd1 F: CTAGCAGAAACAAACCGGGC
mt-Nd1 R: CCGGCTGCGTATTCTACGTT
mt-Rnr2 (16S) F: CCGCAAGGGAAAGATGAAAGAC
*mt-Rnr2 (16S)*R: TCGTTTGGTTTCGGGGTTTC
